# The pyrrolizidine alkaloid lasiocarpine impairs cell cycle progression in vitro

**DOI:** 10.1007/s00204-025-04185-y

**Published:** 2025-09-16

**Authors:** Stefanie Hessel-Pras, Marlena Beckschulte, Antonia Peters, Anja Koellner, Beatrice Rosskopp, Aaron Stahl, Markus Templin, Heike Sprenger, Linda Boehmert, Jan-Heiner Kuepper, Benjamin Sachse, Bernd Schaefer

**Affiliations:** 1 Department of Chemical and Product Safety, German Federal Institute for Risk Assessment, Max-Dohrn-Straße 8-10, 10589 Berlin, Germany; 2 Department of Food and Feed Safety in the Food Chain, German Federal Institute for Risk Assessment, Max-Dohrn-Straße 8-10, 10589 Berlin, Germany; 3 Department of Assay Development, NMI Natural and Medical Sciences Institute, Markwiesenstraße 55, 72770 Reutlingen, Germany; 4 Department of Molecular Cell Biology, Brandenburg University of Technology (BTU) Cottbus-Senftenberg, Postfach 101344, 03013 Cottbus, Germany

**Keywords:** Pyrrolizidine alkaloids, Lasiocarpine, Cell cycle, Genotoxicity

## Abstract

1,2-Unsaturated pyrrolizidine alkaloids (PA) induce severe acute and chronic hepatotoxicity. Effects include an impairment of the cell cycle. To elucidate this effect, cell cycle progression was analyzed by flow cytometry, changes in cell and nucleus size, differential gene expression and protein phosphorylation patterns of regulatory key proteins in the Chinese hamster lung fibroblast cell line V79 and/or the human hepatoblastoma cell line HepG2, both overexpressing human CYP3A4 (V79_3A4_/HepG2_3A4_). Lasiocarpine, a potent PA representative, reduced the cell viability of human CYP3A4-overexpressing cell lines concentration-dependently. Microscopic observation showed a marked increase in cell and nucleus size of V79_3A4_ cells after exposure to 10 µM lasiocarpine. In the human CYP3A4-overexpressing cell lines, cells accumulate in G_2_/M phase after lasiocarpine treatment. Based on these findings, the gene expression pattern of cell cycle-related genes was investigated in HepG2_3A4_ cells showing a decrease of e. g. *WEE1*, and *CHEK1* and an increase of *PAK1* and *ATM*. While results on cell cycle regulation at the level of gene expression are of limited relevance, protein phosphorylation plays an important role. Therefore, we also elucidated the protein phosphorylation status of regulatory key proteins. The results clearly indicate an induction of the DNA damage response and a late G2 arrest. In conclusion, an impairment of the cell cycle was observed. It correlates with the metabolic activation of lasiocarpine and is most likely mediated by adduct formation of the reactive pyrrole esters with DNA, leading to a disruption of cellular homeostasis and genomic instability.

**Supplementary Information:**

The online version contains supplementary material available at 10.1007/s00204-025-04185-y.

## Introduction

Plants produce a wide variety of secondary metabolites of which several function as defense against herbivores and pathogens. In this context, pyrrolizidine alkaloids are suggested to be typical compounds of secondary plant metabolism comprising more than 660 structures known to date (Robertson and Stevens 2017). The ability to synthesize pyrrolizidine alkaloids occurs mainly in the plant families of Asteraceae, Boraginaceae, and Fabaceae (Stegelmeier et al. 1999; Wiedenfeld et al. 2008). While their occurrence has been substantially documented in more than 350 plant species, pyrrolizidine alkaloids are believed to be present in over 6000 plant species based on their chemotaxonomic properties. This would represent at least 3% of all known flowering plants worldwide. However, the content of pyrrolizidine alkaloids can vary considerably depending on the species, plant part, and growing conditions (Flade et al. 2019).

Chemically, pyrrolizidine alkaloids consist of a necine base and one or two necic acids. The necic acids are aliphatic mono- or dicarboxylic acids that can be esterified with the necine base at the C7 and C9 positions. Based on the degree of esterification, pyrrolizidine alkaloids can be classified as monoesters, open diesters, or cyclic diesters. In addition, they are mainly classified according to the structure of their necine base into the platynecine, retronecine, heliotridine, and otonecine type. The retronecine and heliotridine types differ only in their stereoconfiguration at C7. The monocyclic otonecine type is additionally methylated at the nitrogen atom. Due to the presence of a 1,2-double bond in the necine base, which is essential for metabolic bioactivation, the 1,2-unsaturated pyrrolizidine alkaloids, abbreviated as PA in the following, of these three base types are considered to be of particular interest with respect to their toxicological activity. The platynecine type, unlike the other types, has a saturated necine base and is therefore generally considered to be of low toxicological relevance (Fu et al. 2004; Stegelmeier et al. 2016). PA can occur both as their free base and as *N*-oxides. The latter are regarded as to be less relevant in terms of toxicological activity, but can be converted to the free base in the gut of humans and animals.

The adverse effects of PA and their reactive metabolites in experimental animals include hepatotoxicity, developmental toxicity, genotoxicity, and carcinogenicity. They are metabolically activated in the liver by cytochrome P450 monooxygenases (CYP) resulting in reactive electrophilic pyrrol metabolites (Fu et al. 2004; Hessel-Pras et al. 2020). With the exception of monocrotaline, for which CYP2A6 is the major activating CYP isoenzyme, CYP3A4 and CYP3A5 isoenzymes play a dominant role in the formation of reactive (±)-6,7-dihydro-7-hydroxy-1-hydroxymethyl-5H-pyrrolizine (DHP) esters that interfere with cellular macromolecules.

Toxicity in humans is well known from various cases acute of acute poisoning after ingestion of PA containing herbal medicines and teas but also after consumption of grain contaminated with PA containing weeds (Bane et al. 2012; Kakar et al. 2010; Prakash et al. 1999; Schneider et al. 2012; WHO 1988). As early as 1920, frequent cases of human poisoning in South Africa were attributed to the consumption of bread made from wheat flour contaminated with Senecio (Willmot and Robertson 1920). Other representative cases have been documented in Afghanistan, India, and Tajikistan. Symptoms included vomiting, abdominal distention, and severe liver damage, many of which were fatal (EFSA 2017; Mohabbat et al. 1976). When high doses of PA are ingested orally, acute intoxication in humans is manifested by enlargement of the liver (hepatomegaly) accompanied by accumulation of fluid in the abdomen (ascites). High mortality rates are reported as a result of acute liver failure due to hepatocellular necrosis (Stegelmeier et al. 2016). Acute or short-term liver toxicity following oral ingestion of high doses of PA is particularly well known in the form of hepatic veno-occlusive disease (HVOD), or also known as hepatic sinusoidal obstruction syndrome (HSOS). The disease is clinically characterized by abdominal distension, tender hepatomegaly, refractory ascites and jaundice (Hessel-Pras et al. 2020; McGee et al. 1976; Stegelmeier et al. 2016; Stillman et al. 1977; Tandon et al. 1978). Although the picture may be incomplete, the effects at the cellular level are mainly attributed to cross-linking and adduct formation of the pyrrole metabolites with essential cellular macromolecules, including proteins and nucleic acids which impair cellular functions and homeostasis (Stegelmeier et al. 2016; Tábuas et al. 2024). Mutagenicity and tumorigenesis, as observed in in vitro and in animal studies, respectively, are associated with the interaction of reactive electrophilic pyrrol metabolites with the nucleophilic centers of the genomic DNA (EFSA 2011; NTP 2003). Furthermore, PA, its reactive metabolites, but also synthetic PA analogs were attributed as potent antimitogenic agents either in experimental animals and in cell models (Chan 1993). The PA-induced antimitotic effect is associated with the formation of megalocytes commonly observed mainly located in periportal and midzonal regions of the liver or in the kidney of animals.

To gain further insights into the mechanism of cell cycle impairment, we used lasiocarpine as a known highly potent representative of PA in our study. Cell cycle progression was analyzed in thymidine pre-treated cell lines V79 and HepG2 overexpressing the human bioactivating cytochrome P450 monooxygenase isoenzyme CYP3A4 to enhance the bioactivating capability. To further substantiate the view, effects on the transcription of genes involved in cell cycle regulation and on the phosphorylation patterns of proteins known to be involved in cell cycle control were elucidated in the present study.

## Material and methods

### Chemicals

Lasiocarpine in a purity > 95% was purchased from PhytoLab (PhytoLab GmbH & Co. KG, Verstenbergsgreuth, Germany). It was completely dissolved in a mixture of 50% (v/v) water/acetonitrile (ACN), and stored as aliquots of 5 mM stock solutions at − 20 °C until use. Dulbecco´s modified Eagle medium (DMEM) was purchased from Pan-Biotech (Pan-Biotech, Aidenbach, Germany). Standardized fetal bovine serum superior (FBS) was purchased by Sigma Aldrich (Sigma Aldrich, Taufkirchen, Germany) and used for all cell culture experiments. Trypsin/EDTA solution, penicillin/streptomycin solution, and geneticin (G418 sulfate solution, 50 mg/ml) were obtained from Capricorn (Capricorn Scientific, Ebsdorfergrund, Germany), and blasticidin was received by AppliChem (AppliChem GmbH, Darmstadt, Germany). Unless otherwise stated, all other chemicals were purchased from Sigma Aldrich (Sigma Aldrich, Taufkirchen, Germany), Roth (Roth GmbH &Co KG, Karlsruhe, Germany), or Merck (Merck KGaA, Darmstadt, Germany).

### Cell culture

V79 hamster lung fibroblast wild-type (V79_WT_) and human CYP3A4-overexpressing V79 cells (V79_3A4_) were obtained from Hansruedi Glatt (DIfE, Potsdam, Germany). The cells were constructed as described previously (Schneider et al. 1996). HepG2 wild-type (HepG2_WT_) cells were purchased by the European Collection of Cell Cultures (ECACC, Porton Down, UK). Human CYP3A4-overexpressing HepG2 (HepG2_3A4_) were genetically engineered and propagated by Jan-Heiner Kuepper as previously described (Herzog et al. 2015). All cell lines were maintained in DMEM including the respective supplements as summarized in Table 1 at 37 °C in a humidified atmosphere of 5% CO_2_. Cells were subcultured as described previously (Buchmueller et al. 2022; Ebmeyer et al. 2019). All cell lines were cultivated for a maximum of 12 passages after seeding. All experiments were repeated at least 3 times for adequate biological interpretation and statistical analysis.

**Table 1:** Constituents of the culture medium for the cultivation of the cell lines used

Cell line component	HepG2_WT_	HepG2_3A4_	V79_WT_	V79_3A4_
FBS	10%	10%	10%	10%
Penicillin/streptomycin	1%	1%	1%	1%
l-Alanyl-l-glutamine	–	200 mM	–	–
Blasticidin (10 mg/ml)	–	0.03%	–	–
G418 sulfate (50 mg/ml)	–	–	–	1%

Cell cycle synchronization of cell cultures is particularly useful for studying cell cycle regulated events. Several methods have been used to synchronize cell cultures at different stages of the cell cycle. As an inhibitor of DNA synthesis, thymidine can arrest cells at the G_1_/S boundary (Schvartzman et al. 1984). Cells were incubated with 2 mM thymidine for 16 h, followed by 8 h in normal medium and subsequent addition of 2 mM thymidine for further 16 h (Chen and Deng 2018).

### Determination of cell viability by neutral red uptake (NRU) assay

V79 and HepG2 cell lines were seeded into 96-well plates (see Table 2 for cell numbers) in a volume of 100 μl of cell culture medium and cultured at 37 °C in 5% CO_2_. After 24 h after seeding or thymidine pre-treatment, cells were incubated with increasing concentrations of lasiocarpine (0 to 250 μM). Triton X-100 (0.1%) was used as a positive control and medium containing 2.5% ACN as solvent control. Cells were incubated at 37 °C and 5% CO_2_ for 24 h. Subsequently, cells were washed with PBS and 100 µl of neutral red diluted in cell culture medium (40 µg/ml) was added. After incubation for 2 h at 37 °C, the plates were centrifuged at 150 × *g* for 3 min and the supernatant was discarded. Cells were washed twice with PBS and 150 µl of decolorization solution (50% ethanol, 49% deionized water, 1% acetic acid) was added per well and incubated for 10 min at room temperature (RT) in the absence of light. Fluorescence intensity was measured using a Tecan M200Pro microplate reader (Tecan Group Ltd., Männedorf, Switzerland) at an excitation wavelength of 530 nm and an emission wavelength of 645 nm.

**Table 2:** Number of cells seeded for different methods (**bold**, cells pre-treated with thymidine)

Assay	Format	Number of cells/well	
		HepG2	V79
Cytotoxicity (NRU assay)	96-well plate	50,000	6000
**Cytotoxicity (NRU assay)**	**96-well plate**	**12,000**	**2000**
Gene expression analysis	6-well plate	250,000	–
**Flow cytometry**	**12-well plate**	**150,000**	**20,000**
**Microscopic analysis**	**12-well plate**	**50,000**	
**Phosphoproteome**	**75** **cm**^**2**^** flask**	**2,500,000**	–

### Flow cytometry

Cells were seeded in 12-well plates as described. After thymidine treatment, cells were exposed to 10 µM lasiocarpine, 1 µM colchicine as a positive control, or medium containing 0.1% ACN as solvent control. After cell treatment for 0, 2, 4, 6, (15) and 24 h, the supernatant was collected and cells were washed with 500 µl PBS, which was also collected for later flow cytometry analysis. Cells were trypsinized with 200 µl trypsin-EDTA solution per well for 10 min at 37 °C before stopping the reaction by adding 500 µl cell culture medium. Cells were transferred to the respective collected supernatant and centrifuged at 300 × *g* for 3 min. The supernatant was discarded and cells were washed with 700 µl ice-cold PBS before getting centrifuged as described before. Supernatant was discarded and cells were fixed by adding 800 µl of ice-cold methanol for 30 min on ice. After another centrifugation step, the cells were washed with 500 µl PBS, centrifuged and the supernatant was removed again as described above. 100 µl of propidium iodide (PI) staining solution (0.05 mg/ml PI, 10.6 U/ml RNase A in PBS) was added and incubated for 15 min at RT in the absence of light. Cell cycle analysis was performed on a BD accuri C6 flow cytometer using the appropriate software (BD accuri C6).

### Cell staining and microscopic analysis

For quantitative analysis of cell size, microscopic images were captured at 10× magnification using a Zeiss Celldiscoverer 7 high content screening microscope (Zeiss, Jena, Germany). Cells were seeded in 12-well plates and incubated for 24 h. After incubation, cells were washed with PBS and stained with CellMask™ Orange plasma membrane stain (1000× concentration) as a marker of cell boundaries. CellMask™ Orange stock solution was diluted 1:1000 in PBS before adding 0.4 ml per well and incubating at 37 °C for 10 min. After three washes with PBS, the DNA in the nuclei was stained with Hoechst 33,342, a dye that binds preferentially to the AT-rich regions of the DNA. The stock solution (10 mg/ml) was diluted 1:2000 in PBS and 0.4 ml was added per well. After incubation for 10 min at 37 °C, the cells were rinsed with PBS and 0.5 ml pre-warmed DMEM (without phenol red) was added to each well. Six images were taken from each well at different, non-overlapping positions. These images were quantified using Zeiss ZEN 3.1 (blue edition) software. CellMask™ Orange staining was used to define the cell area and Hoechst 33,342 staining for the nuclear area. A watershed algorithm was used to subdivide the nuclear areas. To exclude potential micronuclei, the minimum nucleus size was set to 60. Additionally, a minimum cell size was set to 200 and all cell areas without nuclear area were excluded. The exported data were further processed. Cell areas with multiple nuclei were divided by their number to calculate single cell area. Results are presented as mean ± standard deviation from 140 to several hundred single cells from different biological replicates.

To further investigate the changes in cell size after incubation with lasiocarpine, representative confocal laser scanning microscopy images were taken with a Zeiss LSM 700 in a magnification of 40×. Cells were seeded in 12-well plates on glass coverslips and incubated for 24 h. First, cells were fixed by adding 0.5 ml of 3.7% formaldehyde in medium per well to rinsed cells and incubated at 37 °C for 20 min. The cells were then washed twice with PBS, permeabilized with 0.5 ml of 0.2% Triton X-100 in PBS, and incubated on a shaker for 10 min. ActinGreen™ 488 ReadyProbes™ reagent (AlexaFluor™ 488 phalloidin) was then used to stain F-actin to visualize cytoskeletal organization and overall cell size and shape. Two drops of the reagent were added per ml of PBS, and 0.5 ml was added per well. The cells were incubated for 30 min on an orbital shaker protected from light. After washing the cells three times in PBS, 0.5 ml of a DAPI (4′,6-diamidino-2-phenylindole) solution (3 µM dissolved PBS) was added per well and incubated for 5 min under exclusion of light. Finally, the cells were washed 3 times in PBS before the coverslips were removed from the wells and placed on microscope slides covered with Kaiser’s glycerol gelatine.

### Expression analysis of cell cycle regulatory genes

#### RNA isolation

For gene expression analysis, HepG2_WT_ and HepG2_3A4_ cells were treated with 10 µM lasiocarpine for different times (1, 2, 4, 6, 24, or 48 h). Cells were washed twice with PBS before RNA isolation. Total RNA was isolated using the RNeasy Mini Kit (Qiagen, Hilden, Germany) including an on-column DNase digestion step (RNase-free DNase Set, Qiagen, Hilden, Germany) according to the manufacturer’s protocol. After isolation, RNA concentrations were determined using the Tecan Infinite^®^ 200 PRO Nanoquant platform, a full-spectrum absorbance-based spectrophotometer.

#### Gene expression analysis: cDNA synthesis and quantitative real-time PCR

A maximum of one microgram of RNA was reverse-transcribed into cDNA using the High-Capacity cDNA Reverse Transcription Kit with RNase Inhibitor (Applied Biosystems, Foster City, USA) according to the manufacturer´s instructions. Quantitative real-time PCR (qPCR) was performed using SYBR Green (Maxima SYBR Green/ROX qPCR Master Mix (2×), Fisher Scientific GmbH, Nidderau, Germany), 300 nM of each primer (see Table 3 for sequences) and 1 μl cDNA per sample in a total volume of 10 μl. The thermal cycling program included an initial denaturation step at 95 °C for 15 min, followed by 40 cycles of denaturation at 95 °C for 15 s and annealing and extension at 60 °C for 1 min, and final extension at 60 °C for 15 min. At the end of the run, a dissociation curve analysis was performed. The 2^−ΔΔCt^ method was used for relative quantification of mRNA content (Livak and Schmittgen 2001). Ct values were normalized to the housekeeping gene *GAPDH* and referred to expression values of solvent-treated HepG2_WT_ or HepG2_3A4_ cells at the respective time point.

#### DigiWest protein profiling

A cell number of 2.5 × 10^6^ HepG2_3A4_ cells was seeded in a 75 cm^2^ cell culture flask and cultured at 37 °C in 5% CO_2_. Cells were treated with thymidine as described earlier in this chapter. For analysis of the phosphorylation status of cell cycle-associated proteins, cells were incubated with 10 µM lasiocarpine, with 0.1% ACN serving as solvent control for 0, 6 and 24 h. Cells were then washed twice with ice-cold PBS before harvesting by scraping the cells on ice. After centrifugation at 700 ×* g* for 3.5 min and discarding the supernatants, cell pellets were stored at − 80 °C until analysis.

For cell lysis, 150 µl of lysis buffer (LDS Lysis Buffer, Life Technologies, Carlsbad, CA, USA) supplemented with 10% reducing agent (Thermo Fisher Scientific, Waltham, MA, USA), 4% protease inhibitor and 10% phosphatase inhibitor (both Roche Diagnostics GmbH, Mannheim, Germany) were added to cell pellets on ice. Proteins were denatured by heating to 95 °C for 10 min before transfer to QiaShredder reaction tubes (Qiagen, Hilden, Germany). After centrifugation (16,000 ×* g*, 5 min, RT), eluates were stored at − 80 °C until further use.

Protein quantification was performed by in-gel staining. 1 µl of each original lysate was diluted 1:10 (v/v) in lysis buffer. 10 µl of each aliquot was loaded onto a NuPAGE 4–12% bis–tris precast gel (Thermo Fisher Scientific) and run according to the manufacturer´s instructions. The gel was washed with water and proteins were stained with BlueBandit (VWR, Darmstadt, Germany) for 1 h. The gel was de-stained overnight with ddH_2_O before detection at a LI-COR instrument (LI-COR, Bad Homburg, Germany). Analysis and protein quantification were performed using ImageStudio.

DigiWest was performed as published (Treindl et al. 2016). In brief, 12 μg of cellular protein was loaded onto a SDS–polyacrylamide gel and size-separated using the commercial Invitrogen NuPAGE system (Thermo Fisher Scientific, Waltham, MA, USA). The size-separated proteins were blotted onto a PVDF membrane and biotinylated on the membrane with NHS-PEG12-biotin (50 µM in PBS-T) for 1 h. After washing with PBS-T and drying of the membrane, the individual sample lanes were cut into 96 strips of 0.5 mm width using an automated cutting plotter (Silhouette America, West Orem, UT, USA) each corresponding to a defined molecular weight fraction. Each strip was placed in one well of a 96-well plate and 10 µl of elution buffer (8 M urea, 1% Triton X-100 in 100 mM Tris–HCl pH 9.5) was added. The eluted proteins were diluted with 90 μl of dilution buffer (5% BSA in PBS, 0.02% sodium azide, 0.05% Tween-20) and each of the protein fractions was incubated with one distinct magnetic color-coded bead population (Luminex, Austin, USA) coated with neutravidin. The biotinylated proteins bind to the neutravidin beads such that each bead color represents proteins of a specific molecular weight fraction. All 96 protein-loaded bead populations were mixed resulting in reconstitution of the original lane. Aliquots of the DigiWest bead mixes (about 1/200th per well) were added to 96-well plates containing 50 µl of assay buffer (Blocking Reagent for ELISA, Roche, Rotkreuz, Switzerland) supplemented with 0.2% milk powder, 0.05% Tween 20, and 0.02% sodium azide). Beads were briefly incubated in assay buffer and the buffer was discarded. Primary antibodies (complete list can be found in Supplemental Information, Table S1) were diluted in assay buffer and 30 μl per well were added. After overnight incubation at 15 °C, the bead mixtures were washed twice with PBS-T and species-specific phycoerythrin (PE)-labeled secondary antibodies (Dianova, Hamburg, Germany) were added and incubated at 23 °C for 1 h. Beads were washed twice with PBS-T prior to readout on a Luminex FlexMAP 3D. For quantification of the antibody-specific signals, an Excel-based analysis tool was employed (Treindl et al. 2016) that automatically identifies peaks of appropriate molecular weight and calculates the peak area (reported as accumulated fluorescence intensity/AFI). Signal intensity was normalized to the total amount of protein loaded onto one lane.

### Statistical analysis

Statistical analysis was done with R (version 4.2.2, (R Core Team; 2021). If not otherwise indicated in the respective figure legend, significant differences of treatment groups in comparison to the solvent controls as well as in dependence of incubation time and cell line were tested with three-way ANOVA analysis followed emmeans (estimated marginal means) test as post hoc test with R packages "tidyverse" (version 2.0), "rstatix" (version 0.7.2) and "ggpubr" (version 0.6.0). p values were Benjamini–Hochberg-adjusted for multiple testing and statistical significance is indicated with */^#^ p < 0.05**/^##^p < 0.01***/^###^p < 0.001.

The software package MEV 4.9.0 was used for statistical analysis of protein phosphorylation signals (Saeed et al. 2003) along with Graph Pad Prism (version 9.0.0). Heatmaps of log2-transformed median ratios were created with the R package “ComplexHeatmap” (version 2.14.0) and hierarchical clustering was performed using Euclidian distance and complete linkage. Wilcoxon rank-sum test was used for comparison between groups using the accumulated fluorescence intensity (AFI) values unless stated otherwise. A p value of p < 0.05 was considered as significant.

## Results

### Overexpression of the human cytochrome P450 monooxygenase isoenzyme CYP3A4 enhances the ability of cell lines to bioactivate lasiocarpine

**Fig. 1:**
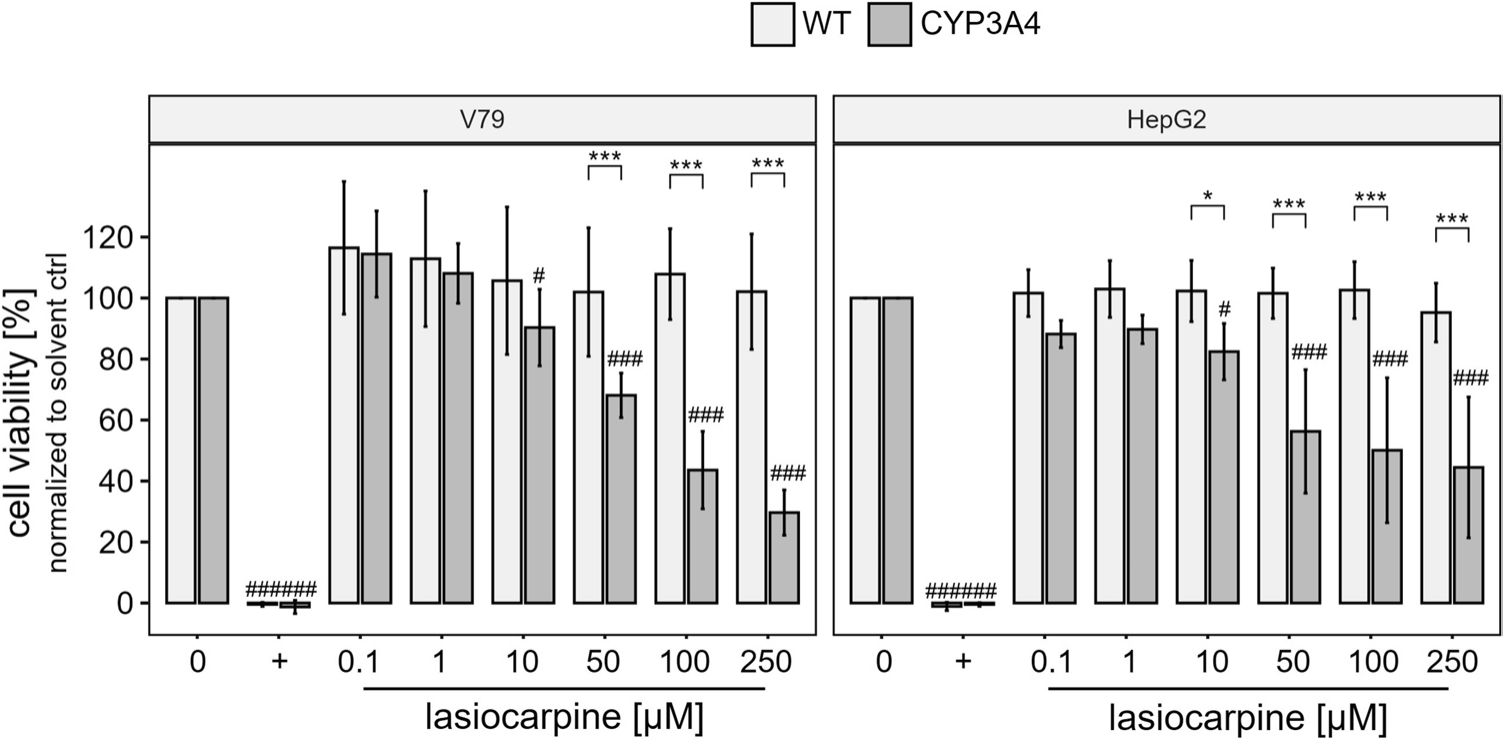
Cell viability measured in thymidine-treated cultures of V79_WT_ and HepG2_WT_ cell lines and their respective derivatives overexpressing human CYP3A4 incubated with lasiocarpine for 24 h. Cells were treated with solvent (0.1% ACN, indicated by “0” in the figure), 0.01% Triton X-100 ( +), and 0.1, 1, 10, 50, 100, or 250 μM lasiocarpine. Cell viability was determined by the neutral red uptake (NRU) assay. Means ± SD are shown from three single experiments with statistical significance levels compared to solvent control indicated by ^#^*p* < 0.05, ^##^*p* < 0.01, ^###^*p* < 0.001 and comparison between both cell lines is indicated by *p < 0.05, **p < 0.01, ***p < 0.001. Significance was tested by three-way ANOVA analysis followed by the estimated marginal means test as a post hoc test. Cell viability data of cells not treated with thymidine are presented in the Supplemental Information Figure S1

In many cultured cell lines, the activity of cytochrome P450 monooxygenases (CYP) is reduced. Consequently, cells lacking the activity of those CYP isoenzymes, that are mainly involved in the bioactivation of PA, (geno)toxic effects upon PA exposure have usually not been observed. In this study, we therefore used derivatives of the cell lines V79 and HepG2 overexpressing human CYP3A4 the isoenzyme that plays a dominant role in the formation of reactive DHP esters from most PA. As indicative for the capability of the cell lines to bioactivate lasiocarpine, we measured cytotoxicity after treatment with lasiocarpine in reference cell lines (V79_WT_ and HepG2_WT_) compared to their derivatives overexpressing human CYP3A4 (V79_3A4_ and HepG2_3A4_) using the neutral red uptake (NRU) assay. Furthermore, we used cell cultures that were treated with thymidine prior to incubation with lasiocarpine (Fig. 1). Treatment with thymidine was performed to collect cells in one cell cycle status based on the allosteric regulation of RNR enzyme in the presence of elevated dTTP concentrations resulting in an arrest of cells at the G_1_/S boundary (Schvartzman et al. 1984) followed by a subsequent reversion of the block by removal of the thymidine prior to cell treatment with lasiocarpine.

While no cytotoxic effect was observed in either wild-type cell lines after lasiocarpine exposure, a significant decrease in cell viability was detected in thymidine-treated cultures of both, HepG2_3A4_, and V79_3A4_ cells, with increasing lasiocarpine concentrations. We derived EC_50_ values of 100 µM and 89 µM in cultures of HepG2_3A4_ and V79_3A4_, respectively. To determine whether an additional cell treatment with thymidine has an effect on cell viability, the induction of cytotoxicity by lasiocarpine was also analyzed without thymidine pre-treatment. However, no significant differences were found in the induction of cytotoxicity by lasiocarpine compared to thymidine pre-treated cell cultures. (Figure S1 in the Supplemental Information).

The data on cytotoxicity were also used to identify non- and sub-toxic concentrations for follow-up studies. Based on these results, a maximum concentration of 10 µM was used for the subsequent experiments using the HepG2_WT_ and V79_WT_ cell lines and the derived overexpressing human CYP3A4 cell lines.

### Lasiocarpine increases cell and nucleus sizes in cell lines overexpressing human CYP3A4

**Fig. 2:**
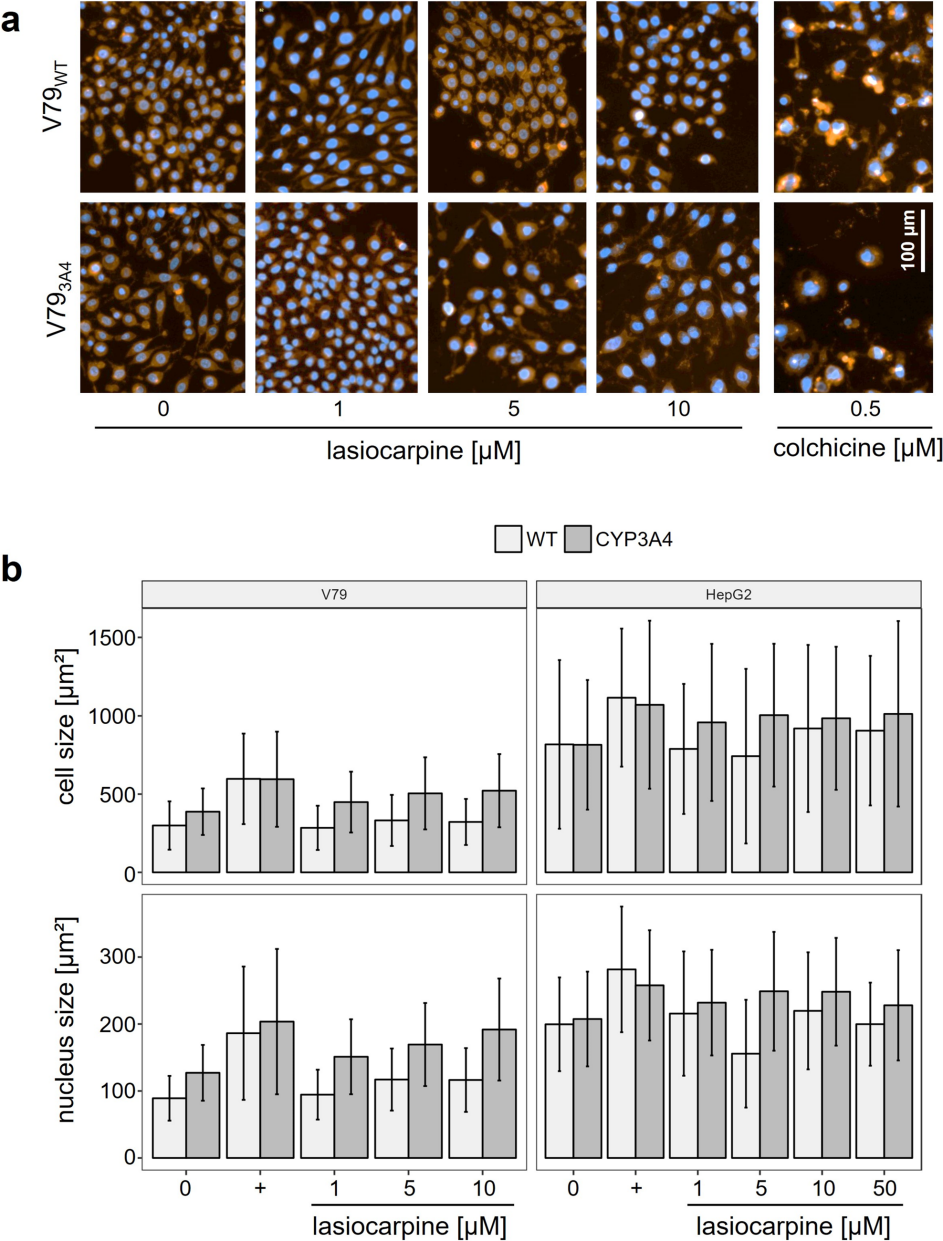
Effects of lasiocarpine treatment on cell and nuclear sizes of V79 and HepG2 cells and their respective derivatives overexpressing human CYP3A4 using the Celldiscoverer 7 high content screening microscope. Cells were seeded as described in the Material and Method section. After treatment with lasiocarpine (1 to 50 µM) for 24 h, nuclei, cell membranes were stained with Hoechst 33,342, or CellMask™ Orange, respectively. **a** Examples of images of V79 cells taken by Celldiscoverer 7 high content screening microscope. **b** Quantitative analysis of cell and nucleus size using Zeiss ZEN 3.1 (blue edition) software. Mean and SD of 140 to several hundred single cells from at least two biological replicates are shown. 0, solvent control (0 µM); + , positive control (0.5 µM colchicine)

To obtain more detailed information on PA-induced effects on cell morphology, V79 and HepG2 wild-type cells and their derivatives overexpressing human CYP3A4 were analyzed in a Celldiscoverer 7 high content screening microscope and by confocal microscopy after treatment with lasiocarpine. Cultures of each cell line were first treated with thymidine, prior exposure to 1, 5, and 10 µM of lasiocarpine. The plasma membranes of the cells were then stained with CellMask™ Orange to define the cell area and with Hoechst 33,342 to define the nuclear area. Cell and nuclear sizes were then quantified using the Celldiscoverer 7 high content screening microscope with Zeiss ZEN 3.1 (blue edition) software (Fig. 2a/b).

As subjectively assessed, treatment of V79_3A4_ cells with lasiocarpine resulted in a significant increased cell size. The effect seems to be more pronounced at higher lasiocarpine concentrations (Fig. 2a/b). Quantitative evaluation of cell and nuclear size after treatment of cells overexpressing human CYP3A4 with lasiocarpine demonstrated a substantial increase in both cell and nucleus size in V79 cells (Fig. 2B) but was not observed in the corresponding HepG2 cell line. As expected, treatment of cells with colchicine as control, which is known to prevent microtubule assembly, showed an increase in cell and nucleus size in all cell lines examined. Images of the HepG2 cell lines are shown as examples in the Supplemental Information (Figure S2).

**Fig. 3:**
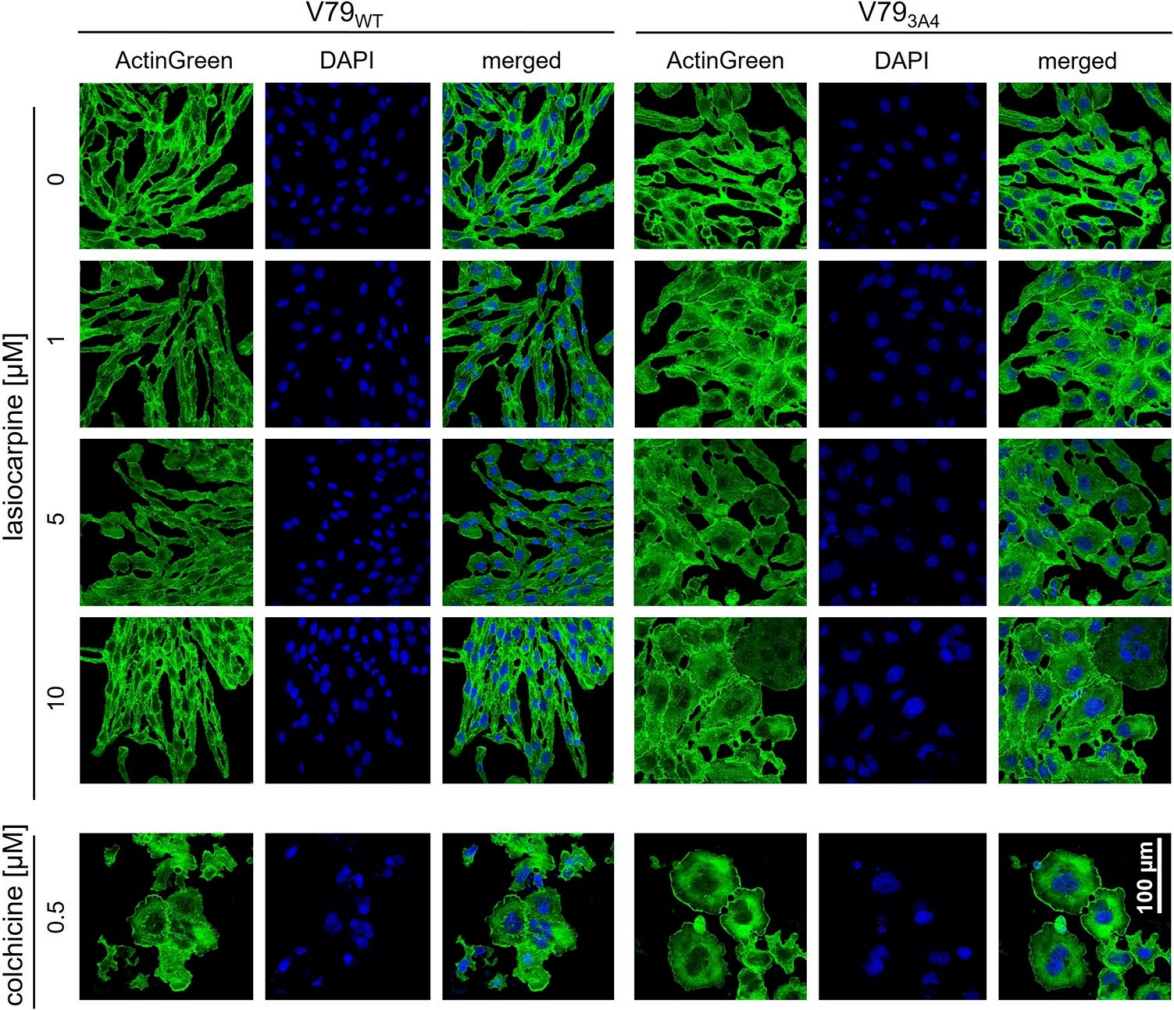
Representative confocal laser scanning microscopy images of V79_WT_ and V79_3A4_ cells. After treatment with thymidine as described in Material and Method section, cells were incubated with 1, 5, or 10 µM lasiocarpine. As controls, cells were treated with solvent (0.1% ACN/ 0 µM), or 0.5 μM colchicine as positive control. Cells were fixed on glass coverslips, permeabilized and stained with AlexaFluor™ 488 phalloidin and DAPI as described in detail in the Material and Method section. Images were taken with Zeiss LSM 700 at 40× magnification

For a further, more detailed look at the cell morphology and cellular organization, we visualized the cytoskeletal organization and overall cell size and shape in lasiocarpine-treated V79_WT_ and V79_3A4_ cells by staining of F-actin with ActinGreen™ 488 ReadyProbes™ reagent (AlexaFluor™ 488 phalloidin) and by visualizing the nuclear DNA with DAPI (Fig. 3). Treatment of V79_WT_ cells with lasiocarpine had no effect on cell size compared to solvent control cells regardless of the lasiocarpine concentration applied. In contrast to V79_WT_ cells, V79_3A4_ cells exposed to lasiocarpine showed increased cell volume. The effect was clearly apparent in cells exposed to 1 µM lasiocarpine and was further enhanced at higher concentrations. In addition, micronuclei formation was observed, especially after cell treatment with 10 µM lasiocarpine. The same trends were observed in HepG2_3A4_ cells although not nearly as pronounced as in V79_3A4_ (Figure S3 in the Supplemental Information). The cells treated with lasiocarpine resemble those obtained after treatment with colchicine (0.5 µM), indicating the potential of lasiocarpine to affect mitosis.

### Exposure to lasiocarpine results in accumulation of cells in G_2_/M phase

In the literature, PA-induced cell cycle arrest has been reported to occur in the S and/or G_2_/M phase (Abdelfatah et al. 2022; Buchmueller et al. 2022; Samuel and Jago 1975). To learn about the lasiocarpine-induced effects on cell cycle progression, V79_WT_ and HepG2_WT_ cells and their respective derivatives overexpressing human CYP3A4 were analyzed by flow cytometry in a BD accuri C6 flow cytometer. The stoichiometric nature of PI used here as the classical nucleic acid stain enabled us to quantify of DNA content and, as the classic means of cell cycle analysis, allows us to reveal the distribution of cells in G_1_, S, and G_2_ cell cycle stage or cells in sub-G_1_ cell death stage, respectively.

The percentage of cells in the G_1_, S, or G_2_/M phase was determined. As before, V79_WT_ or HepG2_WT_ cells were compared with its respected derivative overexpressing human CYP3A4. After thymidine treatment, cells were incubated with solvent, 10 μM lasiocarpine or 1 μM colchicine as positive control and harvested at 0, 2, 4, 6, (15), and 24 h post treatment (all data shown in Figure S4).

**Fig. 4:**
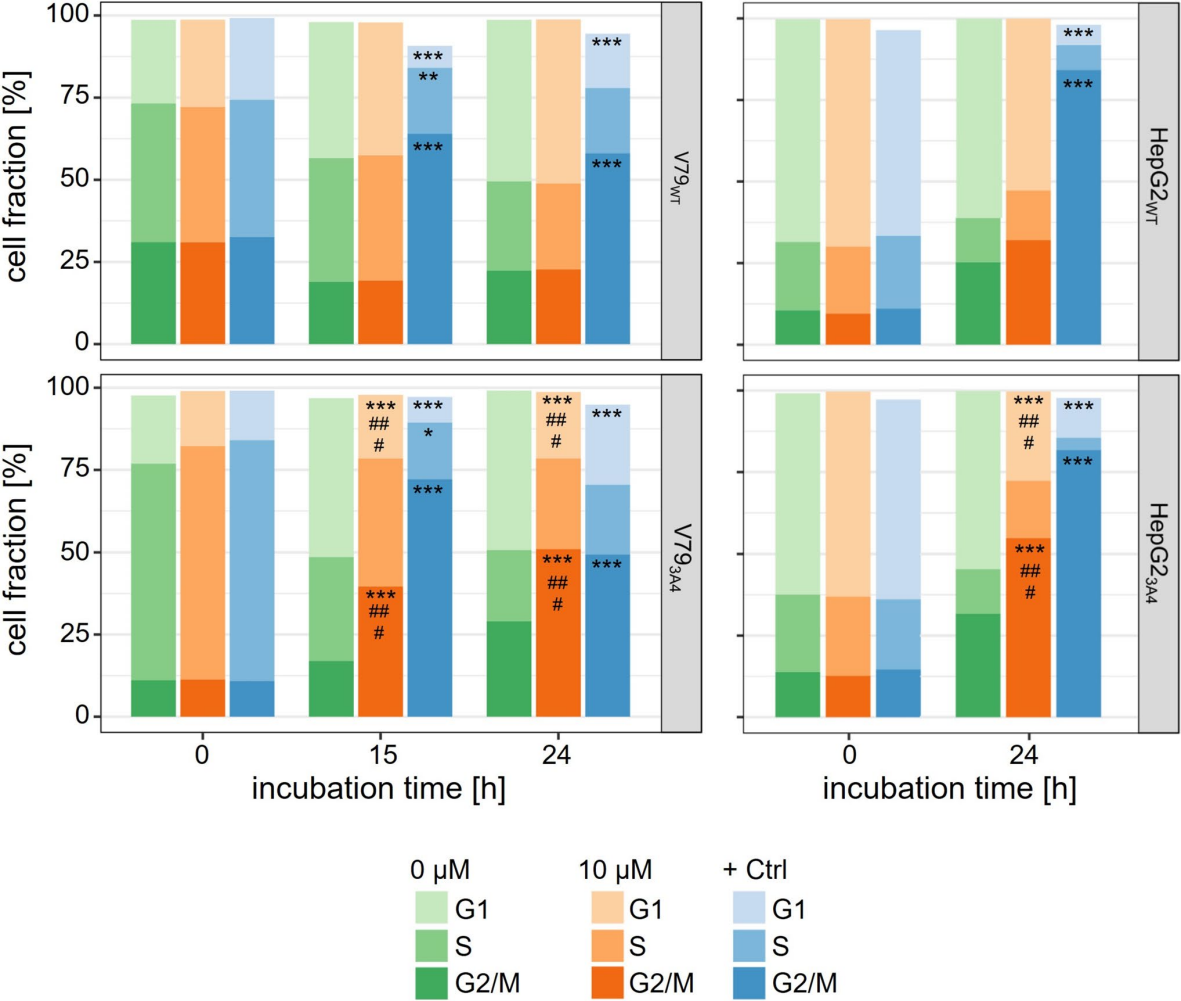
Cell cycle analysis of V79_WT_ and HepG2_WT_ and their respective derivatives overexpressing human CYP3A4 after treatment of cultures with thymidine before exposure to 10 μM lasiocarpine (Lc) for 0, (15), or 24 h. As controls, cells were treated with solvent (0 µM, medium + 0,1% ACN), or 1 μM colchicine (+ Ctrl) as a positive control for cell cycle disruption. Cell cycle analysis was performed by staining of DNA with propidium iodide (PI) and detecting the cell cycle phase by flow cytometry. Cell fraction ad 100% are discarded dead cells. Data are shown as means from three single experiments with statistical significance levels compared to solvent control indicated by *p < 0.05, **p < 0.01, ***p < 0.001 and comparison between both cell lines indicated by ^#^*p* < 0.05, ^##^*p* < 0.01, ^###^*p* < 0.001. Significance was evaluated by three-way ANOVA analysis followed estimated marginal means test as post hoc test

While treatment with colchicine, which inhibits spindle fiber polymerization by binding to free microtubule subunits, induced accumulation of cells in the G_2_/M phase, treatment of wild-type cell lines with lasiocarpine did not induce any measurable shift in the proportions of cells in S and/or G_2_/M phase of the cell cycle. Incubation of V79_3A4_ cells with lasiocarpine resulted in a substantial increase in the number of cells in the G_2_/M phase to 40% after 15 h compared to the untreated control (17%). We observed not only a higher number of cells in G_2_/M phase, but also a slight increase in the number of cells in S phase (39% after 15 h of lasiocarpine treatment vs. 32% in the control, Fig. 4) accompanied with a decrease in the number of cells in G_1_ compared to solvent-treated cells. In HepG2_3A4_ cells exposed to lasiocarpine for 24 h, we measured a significant increase of the cell number in G_2_/M phase. This was accompanied by a significant reduction in the number of cells in the G1 phase compared to the solvent control. The differences to HepG2_WT_ are also significant in this two cell cycle states. In both cell lines, colchicine treatment led to a significant increase in the number of cells in G_2_/M phase and a decrease in G_1_ phase.

### Lasiocarpine induces de-regulation of genes involved in cell cycle regulation in HepG2_3A4_ cells

**Fig. 5:**
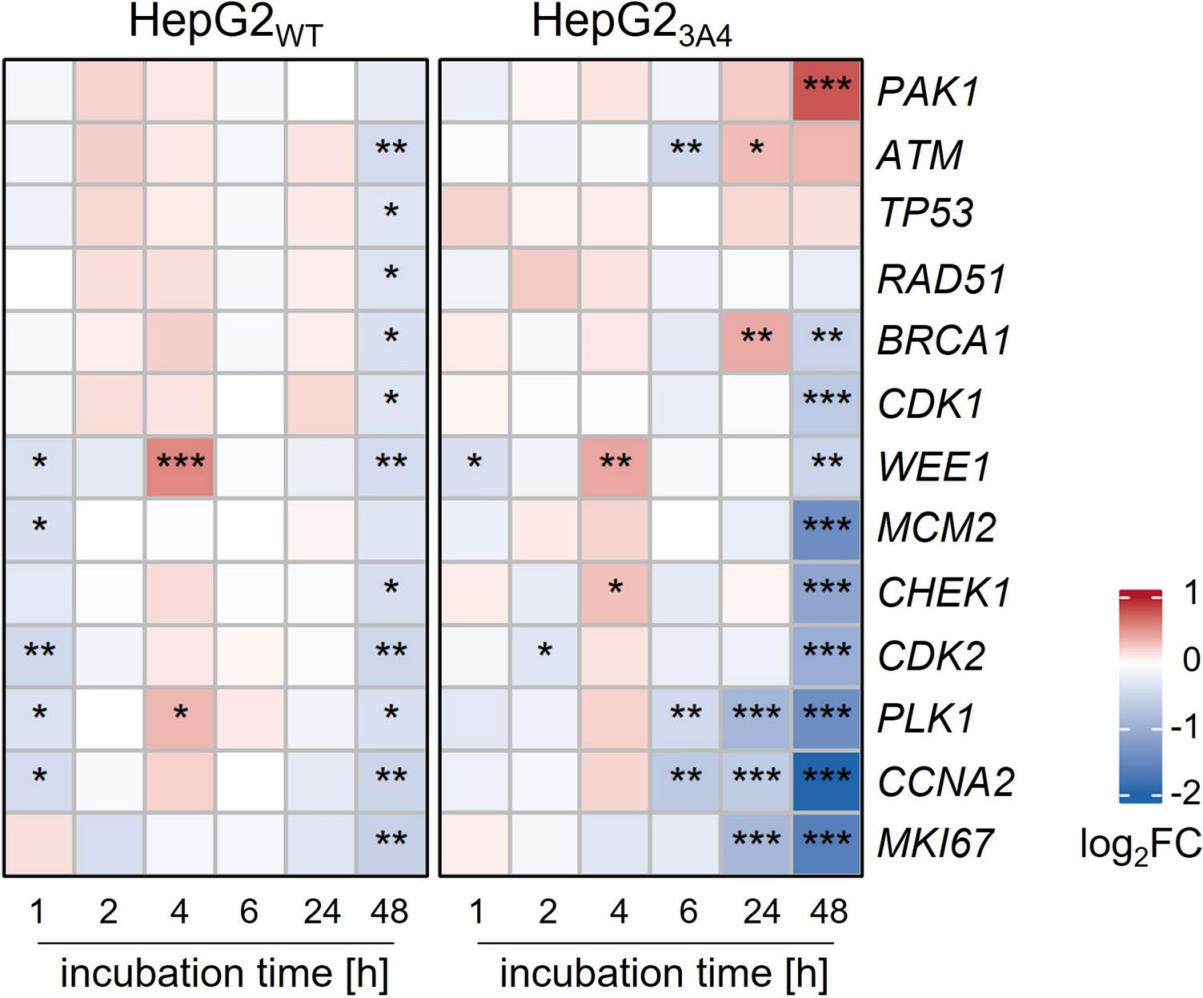
Heat maps representing differential gene expression of various cell cycle regulatory genes in HepG2_WT_ and HepG2_3A4_ cells treated with 10 μM lasiocarpine for 1, 2, 4, 6, 24, or 48 h normalized to the solvent control (0.1% ACN) at the respective time point. Data are presented in log_2_FC (fold change) format. Means are shown from three single experiments with statistical significance levels compared to solvent control indicated by *p < 0.05, **p < 0.01, ***p < 0.001 and comparison between both cell lines indicated by ^#^*p* < 0.05, ^##^*p* < 0.01, ^###^*p* < 0.001. Significance was evaluated by three-way ANOVA analysis followed estimated marginal means test as post hoc test

To obtain information on genes involved in cell cycle regulation, the expression profile of selected candidate genes was elucidated in HepG2_WT_ and in the metabolically competent HepG2_3A4_ cells by quantitative real-time PCR after 1, 2, 4, 6, 24, and 48 h of incubation. Results are shown in Fig. 5. As expected, lasiocarpine-induced effects on the expression of selected candidate genes were more pronounced in HepG2_3A4_ cells compared to HepG2_WT_ cells, at least after 24 and 48 h of incubation. In HepG2_3A4_ cells, a significant downregulation of the transcription of the genes *BRCA1*, *CDC2*, *WEE1*, *MCM2*, *CHK1*, *CDK2*, *PLK1*, *CCNA2*, and *MKI67* was detected after 48 h*.* For the latter three, a significant downregulation was also observed at earlier time points. *BRCA1*, however, was significantly upregulated at an earlier time point of 24 h. The most affected candidate genes were *CCNA2* (fourfold downregulation of gene expression after 48 h of lasiocarpine), followed by *MKI67 (*3.1-fold downregulation). *PAK1*, *ATM*, and *TP53* were (significantly) upregulated after 24 and 48 h of lasiocarpine exposure.

### Lasiocarpine alters the protein phosphorylation status of proteins involved in DNA damage response and cell cycle regulation

The complex molecular network of the cell cycle is mainly controlled by cyclin-dependent kinases (CDK), which promote cell cycle transitions by phosphorylating cell cycle effector proteins as specific downstream targets, making gene expression analysis only partially informative. Therefore, we additionally elucidated the protein phosphorylation status of key regulators in cell cycle control pathways. The protein phosphorylation pattern was examined with a focus on proteins involved in cell cycle regulation and DNA damage response. For this purpose, cultures of HepG2_3A4_ cells were exposed to 10 µM lasiocarpine for 6 or 24 h and cell lysates were analyzed for selected protein phosphorylations (full list in the Supplemental Information Table 1).

**Fig. 6:**
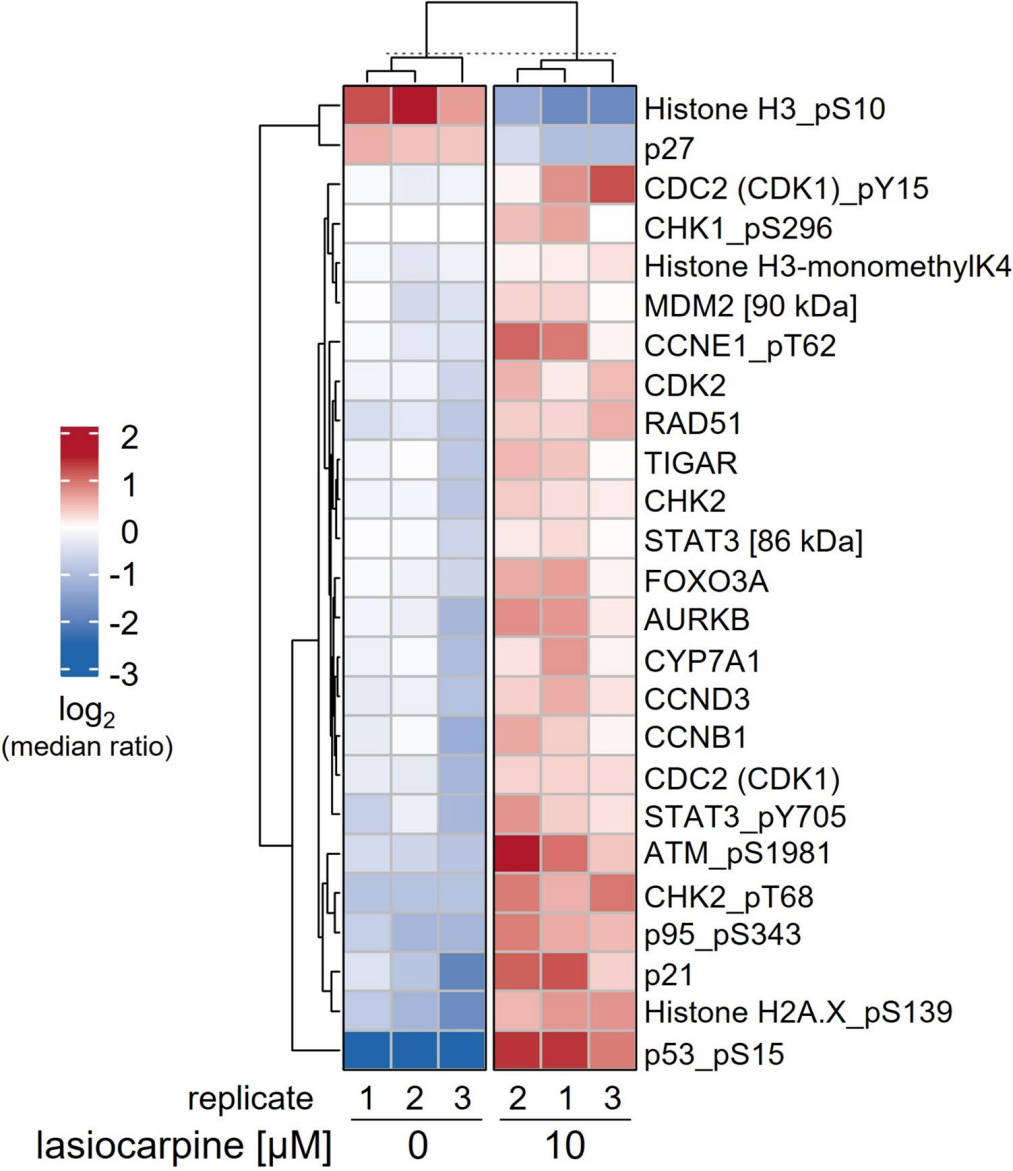
Lasiocarpine-induced changes in protein phosphorylation pattern analyzed by the DigiWest profiling assay. Thymidine pre-treated HepG2_3A4_ cells were incubated with 10 µM lasiocarpine or solvent (0.1% ACN/Ctrl) for 24 h. Samples for protein phosphorylation analysis were prepared and analyzed as described in the Material and Methods section. The heat map shows significant protein phosphorylation status changes (*p* < 0.05) in each biological replicate as log2‑transformed median ratios. Statistical analysis was performed comparing the protein phosphorylation level of solvent-treated (*n* = 3) vs. lasiocarpine-treated cells (*n* = 3) using the Wilcoxon rank-sum test with a significance level set as *p* < 0.05

After 6 h of incubation, very few significant changes in protein phosphorylation status were detected after lasiocarpine treatment compared to the respective untreated control (Supplemental Information, Figure S5). These included p53_pS15, ATM_pS1981, cyclin D3, CHK2_pT68, and cyclin E1 (CCNE1)_pT62. After 24 h of incubation, significantly more altered protein phosphorylations were detected after lasiocarpine treatment compared to solvent-treated cells at the same time. 25 phosphorylation targets were significantly differentially modified compared to control incubations, for example an increase in phosphorylated protein was observed for p53_pS15, ATM_pS1981, CHK2_pT68, H2A.X_pS139 and p21, a significant decrease was observed for p27 and histone H3_pS10 (Fig. 6).

## Discussion

PA have long been known as harmful plant food contaminants. Endogenous xenobiotic metabolism is required to exert PA toxicity in humans and animals. Many studies have shown that lasiocarpine, which we used in this study, is one of the PA that induces the most severe toxic effects, both in vitro and in vivo (Allemang et al. 2018; EFSA 2017). Metabolism and bioactivation of lasiocarpine by human CYP3A4 has been well documented in the literature. Our cell viability data (Fig. 1) confirm the results of a number of studies, e.g., by Ebmeyer et al. (2019), in which a CYP3A4-dependent formation of (geno)toxic metabolites of lasiocarpine was detected, which was accompanied by a reduction of cell viability (Ebmeyer et al. 2019). In animal models, the induction of severe hepatotoxicity was also observed including toxic effects like the formation of cells with an abnormal morphology. After incubation of cultured bovine kidney epithelial cells with PA (riddelliine, seneciphylline, senecionine, and retrorsine), Kim et al. (1993) observed not only a dose-dependent inhibition of colony formation (50–300 μM), but also an increase in the frequency of cells with an altered morphology at a concentration of 500 μM PA. These megalocytes are characterized by an increased cell size with a thin and stretched cytoplasm, accompanied by moderate karyomegaly (Kim et al. 1993). These abnormal cells were also frequently detected in the liver and kidney of PA-exposed animals. In the liver, these megalocytes were mainly observed in periportal and midzonal regions with increasing PA exposure (EFSA 2011; JECFA 2020; Kakar et al. 2010). The enlargement of the volumes of the cytoplasm and the nuclei of hepatocytes is associated with continuous DNA and protein synthesis combined with a lack of cell division in the presence of PA, or more precisely by the respective electrophilic PA metabolites (Bischoff et al. 2018; Jago 1969; Samuel and Jago 1975). The reactive PA intermediates (pyrrole esters) are thought to disrupt the cell cycle by inducing DNA damage response, leading to mitotic bypass resulting in the formation of megalocytosis (Samuel and Jago 1975). In our study, we were able to observe enlarged cells with markedly increased nucleus size after lasiocarpine treatment in cell lines overexpressing human CYP3A4. The increased cell and nucleus size is much more pronounced in V79 cells compared to HepG2 cells, where only slight trends were seen (Figs. 2 and 3). We assume that this observation might be linked to the ability of HepG2 cells to induce p53-dependent cell cycle arrest, initiating repair processes within the cell. V79 cells have a non-functional p53 protein, which may lead to the accumulation of DNA damage and thus enhance susceptibility toward DNA damaging agents (Chaung et al. 1997).

Several in vitro studies have already demonstrated that PA affect cell cycle progression. After incubation of leukocyte cultures or a bovine kidney epithelial cell line with PA, depressed mitosis (Bick and Brown 1972; Bick and Jackson 1968) or inhibited colony formation attributed to mitotic inhibition as the primary cause (Hincks et al. 1991) was observed. In addition, cell cycle arrest induced by PA was also demonstrated (Mattocks and Legg 1980; Thomas et al. 1998; Wilson et al. 2000), as well as an interphase block in late S or early G_2_ phase induced by lasiocarpine (Samuel and Jago 1975). Thomas et al. (1998) were able to show a concentration-dependent response in cell cycle arrest. PA at a low concentration (5 µg/ml) induced S phase delay followed by G_2_/M phase arrest, whereas a high concentration (34.5 μg/ml) resulted in S phase arrest (Thomas et al. 1998). The cell cycle analysis of Buchmueller et al. (2022) revealed a S phase arrest and a decrease in the number of cells in G_1_ phase after incubation with PA of the diester type. Further investigations showed an increase in the number of cells in the S phase accompanied by a decrease in the number of cells in the G_1_/G_0_ phase in HepG2_3A4_ cells (Buchmueller et al. 2022), supporting their previous findings. Abdelfatah et al. (2022) used the same cell line and observed a PA-induced cell cycle arrest in the S phase. Furthermore, these authors detected an abnormal alignment of chromosomes during metaphase after PA treatment, known as a defect of chromosome congression (Abdelfatah et al. 2022). All these findings support the assumption that PA interfere with cell division. Furthermore, micronuclei were detected in HepaRG, HepG2 and V79, both cell lines overexpress human CYP3A4 (Allemang et al. 2018; Buchmueller et al. 2022; Rutz et al. 2020). The detection of centromeres was attributed to an aneugenic origin (Buchmueller et al. 2022).

The cell cycle is a tightly regulated process that requires the interaction of a plethora of proteins. Some of these steps are well understood, such as the interaction between CDKs and their binding partners (cyclins), which is critical for cell division (Morgan 1997). However, there are gaps in our knowledge regarding PA-mediated effects on the cell cycle, leading to cell cycle arrest, megalocytosis, and micronuclei formation. Therefore, in our study, we investigated cell cycle regulation at the transcriptional level, and complemented these data with information on protein phosphorylation pattern of cell cycle regulatory proteins in the presence or absence of PA. For proper functionality of the cell cycle, the cell has specific checkpoints, a mechanism by which the cell actively halts progression through the cell cycle until it can ensure that an essential process, such as DNA replication or mitosis, is completed (Hartwell and Weinert 1989). In addition, the activity of cell cycle-associated proteins must be tightly regulated to ensure a precisely timed one-way transition through the cell cycle. Data from gene expression analysis and the analysis of protein phosphorylation patterns in HepG2_3A4_ cells show a clear effect of PA on cell cycle progression.

A number of (significant) de-regulations were detected at the transcriptional level. After 24 and 48 h of incubation, a slight up-regulation was detected for the genes *ATM* and *TP53. BRCA1* was significantly upregulated at 24 h, but downregulated at 48 h. The proteins of these genes are involved in the induction of cell cycle arrest in the G_2_/M phase and represents a DNA damage response. BRCA1 is required for the activity of the cell cycle checkpoint in S and G_2_/M phase (Wu et al. 2010) as well as for ATM- and ATR-dependent phosphorylation of p53, which in turn mediates cell cycle arrest, allowing for DNA repair or triggering apoptosis when DNA damage is too severe to recover (Hafner et al. 2019). In addition, the expression of polo-like kinase 1 (*PLK1*) gene was significantly reduced after 6, 24, and 48 h of lasiocarpine treatment in HepG2_3A4_ cells. PLK1 plays a key role in the progression of mitosis at the G_2_/M checkpoint. It enables mitotic entry by activating the cyclin-dependent kinase 1 (CDK1)–cyclin B complex and mediates spindle and centromere formation (Sanhaji et al. 2014). Consequently, the absence or the inhibition of PLK1 is associated with impaired replication (Song et al. 2018). Other studies describe the same effect, supporting our findings: PLK1 was one of the most downregulated genes in HepG2_3A4_ cells treated with five different PA in a study conducted by Abdelfatah et al*.* (Abdelfatah et al. 2022). Therefore, the authors concluded that PLK1 is a driver for the subsequent disruption of cell cycle and DNA damage repair signaling cascades. The expression of *CHK1* and *WEE1* was (significantly) upregulated after 4 h of lasiocarpine treatment in both, HepG2_WT_ and HepG2_3A4_ cells. However, incubation for 48 h resulted in a decrease in gene expression, probably as a negative feedback mechanism. The gene *WEE1* encodes a G_2_ checkpoint kinase that plays a critical role in cell cycle regulation and DNA damage detection and repair as part of the DNA damage response pathways (Smith et al. 2020). Abdelfatah et al. (2022) identified CHK1 signaling as significantly inhibited upon treatment with high doses of lasiocarpine, riddelliine, and monocrotaline in HepG2_3A4_ cells (Abdelfatah et al. 2022). The protein CHK1 mediates arrest at cell cycle checkpoints. Specifically, CHK1 was shown to induce cell cycle arrest at both, the S and G_2_ checkpoints. It is activated by ATM/ATR-mediated phosphorylation. Activated CHK1 phosphorylates and, thus, inactivates a protein phosphatase (CDC25), preventing the activation of its downstream target, the CDK1 kinase, which is responsible for the G2/M transition (Xiao et al. 2005).

Importantly, cell cycle progression is largely affected by the phosphorylation of relevant proteins. Thus, changes of proteins on a transcriptional level do only partly reflect the complex regulation. Therefore, in this study, phosphorylation patterns of proteins associated with cell cycle regulation and DNA damage response were also examined in the cell line HepG2_3A4_. The results of protein phosphorylation analysis clearly showed that exposure to lasiocarpine leads to a DNA damage response and an arrest of the cell cycle in G_2_/M. These results underline and expand the above-described gene expression data. Analysis of the protein phosphorylation status revealed a strong increase of phosphorylated histone H2AX at Ser139 (γH2AX) after lasiocarpine treatment, which has already been described in the literature for PA. For example, Buchmueller et al. (2022) and Louisse et al. (2019) observed histone phosphorylation when evaluating a number of PA in HepG2_3A4_ or HepaRG cells (Buchmueller et al. 2022; Louisse et al. 2019). γH2AX phosphorylation primarily represents a response to DNA double-strand breaks (Chen et al. 2010). DNA double-strand breaks can occur when replication forks encounter DNA lesions or repair intermediates (Cannan and Pederson 2016). The observation that micronucleus formation was enhanced in the presence of lasiocarpine in our study can be drawn back to the induction of DNA double-strand breaks promoted by the PA. The formation of γH2AX can also be the result of general DNA damage, that occurs in apoptotic cells. However, the concentration of lasiocarpine in our study, which is in the sub-toxic range, is unlikely to induce apoptotic effects that would explain the strong increase in H2AX phosphorylation observed.

The DNA damage responses are coordinated by several signaling pathways, the most important of which are ATM-CHK2/CHK1 and ATR-CHK1. Activation of these pathways, which is controlled via the phosphorylation status, is critical for proper checkpoint coordination and DNA repair processes, but can also modulate other biological functions such as apoptosis or cell senescence. Specifically, autophosphorylation at serine 1981 stabilizes ATM at sites of DNA strand breaks and initiates a DNA damage response (So et al. 2009). Lasiocarpine exposure also leads to an autophosphorylation of CHK1 at Ser296, which has been shown to be relevant in the regulation of the DNA damage-induced checkpoint control by ATR- or ATM-dependent pathway (Okita et al. 2012). ATM-CHK2/CHK1 signaling activates the p53 system. Indeed, phosphorylation of p53 at serine 15, representing stabilization and activation of p53 was observed in our study. This leads to an arrest of the cell cycle, allowing for repair of DNA damage prior to mitosis (reviewed in Smith et al. 2020, 2010)). Consistent with p53 activation is the up-regulation of MDM2. Phosphorylated p53 induces transcription and activation of MDM2, which in turn downregulates p53, representing a negative feedback loop (reviewed in Momand et al. 2000). Further, p53 activation leads to the transcription of pro-apoptotic genes, such as *Puma*, *Noxa*, *BAX*, and *Apaf1*, resulting in apoptotic cell death if the damage is too severe (Bieging et al. 2014). The induction of apoptosis by PA has already been well studied in various liver cell models and also in vivo (Glück et al. 2020; Waizenegger et al. 2018; Yang et al. 2017).

The DNA damage response is inversely linked to cell cycle progression. If DNA damage or incompletely replicated DNA is not sufficiently repaired, cells arrest at the G_2_/M checkpoint and fail to initiate mitosis. Consistent with the activation of the ATM-CHK2-p53 signaling pathway, also a lasiocarpine-induced increase in p21 phosphorylation was observed. The protein p21 is a CDK inhibitor and a major target of p53 activity. p21 inhibits the activity of cyclin/CDK complexes (Harper et al. 1995) and is therefore associated with DNA damage-induced cell cycle arrest (Al Bitar and Gali-Muhtasib 2019). p21-mediated phosphorylation of threonine and tyrosine residues in the G loop (T14 and Y15) of CDK1 inhibits its kinase activity. This phosphorylation, as observed for CDK1 at threonine 15 in our study, does not significantly alter the structure of CDK, but it does reduce its affinity for substrates resulting in a cell cycle arrest (Pellarin et al. 2025).

The importance of the p53 system was also demonstrated by the results obtained in the V79 cell line. This cell line has a mutated p53 sequence containing two point mutations located within a putative DNA-binding domain resulting in a non-functional p53 that affects DNA damage, DNA repair and apoptosis (Chaung et al. 1997). In addition, the G_1_ phase is either absent or very short in these cells as also observed in our cell cycle analysis. The fact that the cell cycle is not sufficiently arrested to repair DNA damage leads to the accumulation of DNA adducts and thus to the enhancement of lasiocarpine-induced (toxic) effects in the cell line V79 overexpressing human CYP3A4. HepG2 cells have an intact p53 system allowing cell cycle arrest to repair DNA damage and the observed toxic (cytological) effects of lasiocarpine appear to be much less pronounced (Table 3).

**Table 3:** Primer sequences of target genes

*Gene*	Forward primer (5′ → 3′)	Reverse primer (5′ → 3′)
*ATM*	AACGCCCTGAATTGAACCCT	GCTTGTGTTGAGGCTGATACA
*BRCA1*	AGGAAACTTGAAACCTGGGCAT	GCTGGTTTCGAACTCCTGAC
*CDK1*	AGCTGGCTCTTGGAAATTGA	GGTATGGTAGATCCCGGCTTA
*CDK2*	CTGGCATTCCTCTTCCCCTC	GGCTTGGTCACATCCTGGAA
*CHEK1*	ATCAACTCATGGCAGGGGTG	TGGGAGACTCTGACACACCA
*CCNA2*	CCCTGCATTTGGCTGTGAAC	GTGTCTCTGGTGGGTTGAGG
*GAPDH*	ATTTGGCTACAGCAACAGGG	CAACTGTGAGGAGGGGAGA
*MCM2*	AGCAGTTAGTGGCAGAGCAG	AGCAGCCTGAATACGCAACT
*MKI67*	GCTTCTCTTCTGACCCTGATG	GGCGTATTAGGAGGCAAGTTT
*TP53*	GGTGCGTGTTTGTGCCTGT	TCCCCTTTCTTGCGGAGATT
*PAK1*	GCCTGACATGATACCCTGCC	AGCAAACATCCCCAACACCC
*PLK1*	GGTGACAGCCTGCAGTACAT	CAGATGCAGGTGGGAGTGAG
*RAD51*	TTACACGTGCCTGTAGTCCC	CAGCAGTGCAATCTCGACTC
*WEE1*	CCACAAGTTGAAGAGGGCGA	GTCACAGTGTTCAGGGGGAG

## Conclusion

In summary, lasiocarpine impairs cell division accompanied by changes in cell volume and nucleus size. This effect is associated with the bioactivation of lasiocarpine by human CYP3A4 into reactive intermediates as shown here in human CYP3A4-overexpressing cell lines. At the molecular level, the affected cells were characterized by altered protein phosphorylation patterns indicative of effects on cell cycle control pathway, and induction of the DNA damage response. The data from our studies clearly point to a DNA damage-induced mode of action associated with effects on G_2_/M cell cycle progression. We propose that damaging the DNA by adduct formation promoted by reactive pyrrole esters as the initial event. The subsequent induction of the DNA damage response pathway leads then to a bypass of mitosis rather than a complete cell cycle block (Prakash et al. 1999). Continued biosynthesis of cellular components might subsequently lead to the formation of megalocytosis characterized by enlarged volumes of cells and nuclei as probably DNA and protein synthesis continues but cell division fails to occur (Bischoff et al. 2018; Jago 1969). Disruption of protein functionality, which may be related to the formation of protein adducts, could also have an impact on the proper progression of cell division but needs to be further investigated.

## Supplementary Information

Below is the link to the electronic supplementary material.

Supplementary material 1

Supplementary material 2

## Abbreviations

ACN: Acetonitrile
AFI: Accumulated fluorescence intensity
ANOVA: Analysis of variance
ATM: Ataxia telangiectasia mutated
AURKB: Aurora kinase B
BRCA1: Breast cancer gene 1
BSA: Bovine serum albumin
CDK1/2,: Cyclin-dependent kinase ½
CHEK1/2: Checkpoint kinase ½
CCNA2/B1/D1/E1: Cyclin A2/B1/D1/E1
GAPDH: Glyceraldehyde-3-phosphate dehydrogenase
CYP: Cytochrome P450 monooxygenase
DHP: (±)-6,7-Dihydro-7-hydroxy-1-hydroxymethyl-5H-pyrrolizine
DMEM: Dulbecco´s modified Eagle medium
FBS: Fetal bovine serum superior
G418: Geneticin
FOXO3A: Forkhead box O3
HSOS: Hepatic sinusoidal obstruction syndrome
HVOD: Hepatic veno-occlusive disease
MCM2: Minichromosome maintenance complex component 2
MDM2: Mouse double minute 2
KI67: Marker of proliferation Ki-67
NRU: Neutral red uptake
p21 p27 (CDKN1): Cyclin-dependent kinase inhibitor 1A
p95 (NBS1): Nibrin
PAK1 p21 (RAC1): Activated kinase 1
PA: 1,2-Unsaturated pyrrolizidine alkaloid
PBS: Phosphate-buffered saline
PI: Propidium iodide
PLK1: Polo-like kinase 1
VDF: Polyvinylidene fluoride
qRT-PCR: Quantitative real-time polymerase chain reaction
RAD51: DNA repair protein RAD51 homolog 1
RT: Room temperature
SD: Standard deviation
STAT3: Signal transducer and activator of transcription 3
TIGAR: TP53-induced glycolysis regulatory phosphatase
TP53/p53: Tumor protein p53
WEE1: Wee1-like protein kinase
WT: Wild type

## References

[CR1] Abdelfatah S, Naß J, Knorz C, Klauck SM, Küpper JH, Efferth T (2022) Pyrrolizidine alkaloids cause cell cycle and DNA damage repair defects as analyzed by transcriptomics in cytochrome P450 3A4-overexpressing HepG2 clone 9 cells. Cell Biol Toxicol 38(2):325–345. 10.1007/s10565-021-09599-9 33884520 10.1007/s10565-021-09599-9 PMC8986750

[CR2] Al Bitar S, Gali-Muhtasib H (2019) The role of the cyclin dependent kinase inhibitor p21(cip1/waf1) in targeting cancer: molecular mechanisms and novel therapeutics. Cancers. 10.3390/cancers11101475 31575057 10.3390/cancers11101475 PMC6826572

[CR3] Allemang A, Mahony C, Lester C, Pfuhler S (2018) Relative potency of fifteen pyrrolizidine alkaloids to induce DNA damage as measured by micronucleus induction in HepaRG human liver cells. Food Chem Toxicol 121:72–81. 10.1016/j.fct.2018.08.003 30125636 10.1016/j.fct.2018.08.003

[CR4] Bane A, Seboxa T, Mesfin G et al (2012) An outbreak of veno-occlusive liver disease in northern Ethiopia, clinical findings. Ethiop Med J 50(Suppl 2):9–16 22946291

[CR5] Bick YA, Brown JK (1972) An analysis of chromosomal sensitivity of HPK1 cells from the marsupial *Potorous tridactylus* to x-rays and to heliotrine. Cytobios 5(19):189–200 5065177

[CR6] Bick YA, Jackson WD (1968) Effects of the pyrrolizidine alkaloid heliotrine on cell division and chromosome breakage in cultures of leucocytes from the marsupial *Potorous tridactylus*. Aust J Biol Sci 21(3):469–481. 10.1071/bi9680469 5664136 10.1071/bi9680469

[CR7] Bieging KT, Mello SS, Attardi LD (2014) Unravelling mechanisms of p53-mediated tumour suppression. Nat Rev Cancer 14(5):359–370. 10.1038/nrc3711 24739573 10.1038/nrc3711 PMC4049238

[CR8] Bischoff K, Mukai M, Ramaiah SK (2018) Chapter 15 - liver toxicity. In: Gupta RC (ed) Veterinary toxicology 3rd ed. Academic Press, pp 239–257

[CR9] Buchmueller J, Enge AM, Peters A et al (2022) The chemical structure impairs the intensity of genotoxic effects promoted by 1,2-unsaturated pyrrolizidine alkaloids *in vitro*. Food Chem Toxicol 164:113049. 10.1016/j.fct.2022.113049 35500694 10.1016/j.fct.2022.113049

[CR10] Cannan WJ, Pederson DS (2016) Mechanisms and consequences of double-strand DNA break formation in chromatin. J Cell Physiol 231(1):3–14. 10.1002/jcp.25048 26040249 10.1002/jcp.25048 PMC4994891

[CR11] Chan P (1993) NTP technical report on the toxicity studies of Riddelliine (CAS no. 23246-96-0) administered by gavage to F344 rats and B6C3F1 mice. Tox Rep Ser 27:1-d9 12209179

[CR12] Chaung W, Mi LJ, Boorstein RJ (1997) The p53 status of Chinese hamster V79 cells frequently used for studies on DNA damage and DNA repair. Nucleic Acids Res 25(5):992–994. 10.1093/nar/25.5.992 9023109 10.1093/nar/25.5.992 PMC146528

[CR13] Chen G, Deng X (2018) Cell Synchronization by Double Thymidine Block. Bio Protoc. 10.21769/BioProtoc.2994 30263905 10.21769/BioProtoc.2994 PMC6156087

[CR14] Chen T, Mei N, Fu PP (2010) Genotoxicity of pyrrolizidine alkaloids. J Appl Toxicol 30(3):183–196. 10.1002/jat.1504 20112250 10.1002/jat.1504 PMC6376482

[CR15] Ebmeyer J, Braeuning A, Glatt H, These A, Hessel-Pras S, Lampen A (2019) Human CYP3A4-mediated toxification of the pyrrolizidine alkaloid lasiocarpine. Food Chem Toxicol 130:79–88. 10.1016/j.fct.2019.05.019 31103741 10.1016/j.fct.2019.05.019

[CR16] EFSA (2011) Scientific Opinion on Pyrrolizidine alkaloids in food and feed - EFSA Panel on Contaminants in the Food Chain (CONTAM). EFSA J 9(11):2406–2540

[CR17] EFSA (2017) Risks for human health related to the presence of pyrrolizidine alkaloids in honey, tea, herbal infusions and food supplements. EFSA J 15(7):34. 10.2903/j.efsa.2017.4908 10.2903/j.efsa.2017.4908 PMC7010083 32625569

[CR18] Flade J, Beschow H, Wensch-Dorendorf M, Plescher A, Wätjen W (2019) Occurrence of nine pyrrolizidine alkaloids in *Senecio vulgaris* L. depending on developmental stage and season. Plants. 10.3390/plants8030054 30841617 10.3390/plants8030054 PMC6473320

[CR19] Fu PP, Xia Q, Lin G, Chou MW (2004) Pyrrolizidine alkaloids—genotoxicity, metabolism enzymes, metabolic activation, and mechanisms. Drug Metab Rev 36(1):1–55. 10.1081/DMR-120028426 15072438 10.1081/dmr-120028426

[CR20] Glück J, Waizenegger J, Braeuning A, Hessel-Pras S (2020) Pyrrolizidine alkaloids induce cell death in human HepaRG cells in a structure-dependent manner. Int J Mol Sci. 10.3390/ijms22010202 33379168 10.3390/ijms22010202 PMC7795836

[CR21] Hafner A, Bulyk ML, Jambhekar A, Lahav G (2019) The multiple mechanisms that regulate p53 activity and cell fate. Nat Rev Mol Cell Biol 20(4):199–210. 10.1038/s41580-019-0110-x 30824861 10.1038/s41580-019-0110-x

[CR22] Harper JW, Elledge SJ, Keyomarsi K et al (1995) Inhibition of cyclin-dependent kinases by p21. Mol Biol Cell 6(4):387–400. 10.1091/mbc.6.4.387 7626805 10.1091/mbc.6.4.387 PMC301199

[CR23] Hartwell LH, Weinert TA (1989) Checkpoints: controls that ensure the order of cell cycle events. Sci 246(4930):629–634. 10.1126/science.2683079 10.1126/science.2683079 2683079

[CR24] Herzog N, Katzenberger N, Martin F, Schmidtke KU, K JH (2015) Generation of cytochrome P450 3A4-overexpressing HepG2 cell clones for standardization of hepatocellular testosterone 6β-hydroxylation activity. J Cell Biotechnol 1:15–26. 10.3233/JCB-15002

[CR25] Hessel-Pras S, Braeuning A, Guenther G et al (2020) The pyrrolizidine alkaloid senecionine induces CYP-dependent destruction of sinusoidal endothelial cells and cholestasis in mice. Arch Toxicol 94(1):219–229. 10.1007/s00204-019-02582-8 31606820 10.1007/s00204-019-02582-8

[CR26] Hincks JR, Kim HY, Segall HJ, Molyneux RJ, Stermitz FR, Coulombe RA Jr (1991) DNA cross-linking in mammalian cells by pyrrolizidine alkaloids: structure-activity relationships. Toxicol Appl Pharmacol 111(1):90–98. 10.1016/0041-008x(91)90137-4 1949039 10.1016/0041-008x(91)90137-4

[CR27] Jago MV (1969) The development of the hepatic megalocytosis of chronic pyrrolizidine alkaloid poisoning. Am J Pathol 56(3):405–421 5822314 PMC2013586

[CR28] JECFA (2020) Safety evaluation of certain food additives and contaminants: prepared by the eightieth meeting of the Joint FAO/WHO expert Committee on food Additives (JECFA). Supplement 2: Pyrrolizidine alkaloids. WHO Food Additives Series, 71-S2

[CR29] Kakar F, Akbarian Z, Leslie T et al (2010) An outbreak of hepatic veno-occlusive disease in Western afghanistan associated with exposure to wheat flour contaminated with pyrrolizidine alkaloids. J Toxicol 2010:313280. 10.1155/2010/313280 20652038 10.1155/2010/313280 PMC2905904

[CR30] Kim HY, Stermitz FR, Molyneux RJ, Wilson DW, Taylor D, Coulombe RA (1993) Structural influences on pyrrolizidine alkaloid-induced cytopathology. Toxicol Appl Pharmacol 122(1):61–69. 10.1006/taap.1993.1172 8378933 10.1006/taap.1993.1172

[CR31] Livak KJ, Schmittgen TD (2001) Analysis of relative gene expression data using real-time quantitative PCR and the 2(-Delta Delta C(T)) Method. Methods 25(4):402–408. 10.1006/meth.2001.1262 11846609 10.1006/meth.2001.1262

[CR32] Louisse J, Rijkers D, Stoopen G et al (2019) Determination of genotoxic potencies of pyrrolizidine alkaloids in HepaRG cells using the gammaH2AX assay. Food Chem Toxicol 131:110532. 10.1016/j.fct.2019.05.040 31154085 10.1016/j.fct.2019.05.040

[CR33] Mattocks AR, Legg RF (1980) Antimitotic activity of dehydroretronecine, a pyrrolizidine alkaloid metabolite, and some analogous compounds, in a rat liver parenchymal cell line. Chem Biol Interact 30(3):325–336. 10.1016/0009-2797(80)90055-1 7379211 10.1016/0009-2797(80)90055-1

[CR34] McGee J, Patrick RS, Wood CB, Blumgart LH (1976) A case of veno-occlusive disease of the liver in Britain associated with herbal tea consumption. J Clin Pathol 29(9):788–794. 10.1136/jcp.29.9.788 977780 10.1136/jcp.29.9.788 PMC476180

[CR35] Mohabbat O, Younos MS, Merzad AA, Srivastava RN, Sediq GG, Aram GN (1976) An outbreak of hepatic veno-occlusive disease in north-western Afghanistan. Lancet 2(7980):269–271 59848 10.1016/s0140-6736(76)90726-1

[CR36] Momand J, Wu H-H, Dasgupta G (2000) MDM2—master regulator of the p53 tumor suppressor protein. Gene 242(1):15–29. 10.1016/S0378-1119(99)00487-4 10721693 10.1016/s0378-1119(99)00487-4

[CR37] Morgan DO (1997) Cyclin-dependent kinases: engines, clocks, and microprocessors. Annu Rev Cell Dev Biol 13:261–291. 10.1146/annurev.cellbio.13.1.261 9442875 10.1146/annurev.cellbio.13.1.261

[CR38] NTP (2003) Toxicology and carcinogenesis studies of riddelliine (CAS No. 23246-96-0) in F344/N rats and B6C3F1 mice (gavage studies). Natl Toxicol Program Tech Rep Ser 508:1–280 12844193

[CR39] Okita N, Minato S, Ohmi E, Tanuma S, Higami Y (2012) DNA damage-induced CHK1 autophosphorylation at Ser296 is regulated by an intramolecular mechanism. FEBS Lett 586(22):3974–3979. 10.1016/j.febslet.2012.09.048 23068608 10.1016/j.febslet.2012.09.048

[CR40] Pellarin I, Dall’Acqua A, Favero A et al (2025) Cyclin-dependent protein kinases and cell cycle regulation in biology and disease. Signal Transduct Target Ther 10(1):11. 10.1038/s41392-024-02080-z 39800748 10.1038/s41392-024-02080-z PMC11734941

[CR41] Prakash AS, Pereira TN, Reilly PEB, Seawright AA (1999) Pyrrolizidine alkaloids in human diet. Mutat Res/genet Toxicol Environ Mutagenesis 443(1–2):53–67. 10.1016/S1383-5742(99)00010-1 10.1016/s1383-5742(99)00010-1 10415431

[CR42] Robertson J, Stevens K (2017) Pyrrolizidine alkaloids: occurrence, biology, and chemical synthesis. Nat Prod Rep 34(1):62–89. 10.1039/c5np00076a 27782262 10.1039/c5np00076a

[CR43] Rutz L, Gao L, Küpper JH, Schrenk D (2020) Structure-dependent genotoxic potencies of selected pyrrolizidine alkaloids in metabolically competent HepG2 cells. Arch Toxicol 94(12):4159–4172. 10.1007/s00204-020-02895-z 32910235 10.1007/s00204-020-02895-z PMC7655576

[CR44] Saeed AI, Sharov V, White J et al (2003) TM4: a free, open-source system for microarray data management and analysis. Biotechniques 34(2):374–378. 10.2144/03342mt01 12613259 10.2144/03342mt01

[CR45] Samuel A, Jago MV (1975) Localization in the cell cycle of the antimitotic action of the pyrrolizidine alkaloid, lasiocarpine and of its metabolite, dehydroheliotridine. Chem Biol Interact 10(3):185–197. 10.1016/0009-2797(75)90112-X 1126005 10.1016/0009-2797(75)90112-x

[CR46] Sanhaji M, Ritter A, Belsham HR et al (2014) Polo-like kinase 1 regulates the stability of the mitotic centromere-associated kinesin in mitosis. Oncotarget 5(10):3130–3144. 10.18632/oncotarget.1861 24931513 10.18632/oncotarget.1861 PMC4102797

[CR47] Schneider A, Schmalix WA, Siruguri V et al (1996) Stable expression of human cytochrome P450 3A4 in conjunction with human NADPH-cytochrome P450 oxidoreductase in V79 Chinese hamster cells. Arch Biochem Biophys 332(2):295–304. 10.1006/abbi.1996.0345 8806738 10.1006/abbi.1996.0345

[CR48] Schneider J, Tsegaye Y, WT M et al (2012) Veno-occlusive liver disease: a case report. Ethiop Med J 50:47–51 22946295

[CR49] Schvartzman JB, Krimer DB, Van’t Hof J (1984) The effects of different thymidine concentrations on DNA replication in pea-root cells synchronized by a protracted 5-fluorodeoxyuridine treatment. Exp Cell Res 150(2):379–389. 10.1016/0014-4827(84)90581-0 6229414 10.1016/0014-4827(84)90581-0

[CR50] Smith J, Tho LM, Xu N, Gillespie DA (2010) The ATM-Chk2 and ATR-Chk1 pathways in DNA damage signaling and cancer. Adv Cancer Res 108:73–112. 10.1016/b978-0-12-380888-2.00003-0 21034966 10.1016/B978-0-12-380888-2.00003-0

[CR51] Smith HL, Southgate H, Tweddle DA, Curtin NJ (2020) DNA damage checkpoint kinases in cancer. Expert Rev Mol Med 22:e2. 10.1017/erm.2020.3 32508294 10.1017/erm.2020.3

[CR52] So S, Davis AJ, Chen DJ (2009) Autophosphorylation at serine 1981 stabilizes ATM at DNA damage sites. J Cell Biol 187(7):977–990. 10.1083/jcb.200906064 20026654 10.1083/jcb.200906064 PMC2806275

[CR53] Song R, Hou G, Yang J et al (2018) Effects of PLK1 on proliferation, invasion and metastasis of gastric cancer cells through epithelial-mesenchymal transition. Oncol Lett 16(5):5739–5744. 10.3892/ol.2018.9406 30405751 10.3892/ol.2018.9406 PMC6202541

[CR54] Stegelmeier BL, Edgar JA, Colegate SM et al (1999) Pyrrolizidine alkaloid plants, metabolism and toxicity. J NatToxins 8(1):95–116 10091131

[CR55] Stegelmeier BL, Colegate SM, Brown AW (2016) Dehydropyrrolizidine alkaloid toxicity, cytotoxicity, and carcinogenicity. Toxins. 10.3390/toxins8120356 27916846 10.3390/toxins8120356 PMC5198550

[CR56] Stillman AS, Huxtable R, Consroe P, Kohnen P, Smith S (1977) Hepatic veno-occlusive disease due to pyrrolizidine (*Senecio*) poisoning in Arizona. Gastroenterology 73(2):349–352 873137

[CR57] Tábuas B, Cruz Barros S, Diogo C, Cavaleiro C, Sanches Silva A (2024) Pyrrolizidine alkaloids in foods, herbal drugs, and food supplements: chemistry, metabolism, toxicological significance, analytical methods, occurrence, and challenges for future. Toxins 16(2):79 38393157 10.3390/toxins16020079 PMC10892171

[CR58] Tandon HD, Tandon BN, Mattocks AR (1978) An epidemic of veno-occlusive disease of the liver in Afghanistan. Pathologic features. Am J Gastroenterol 70(6):607–613 742612

[CR59] R Core Team (2021) R: A Language and Environment for Statistical Computing. Austria, Vienna

[CR60] Thomas HC, Lamé MW, Morin D, Wilson DW, Segall HJ (1998) Prolonged cell-cycle arrest associated with altered cdc2 kinase in monocrotaline pyrrole-treated pulmonary artery endothelial cells. Am J Respir Cell Mol Biol 19(1):129–142. 10.1165/ajrcmb.19.1.2895 9651189 10.1165/ajrcmb.19.1.2895

[CR61] Treindl F, Ruprecht B, Beiter Y et al (2016) A bead-based western for high-throughput cellular signal transduction analyses. Nat Commun 7(1):12852. 10.1038/ncomms12852 27659302 10.1038/ncomms12852 PMC5036152

[CR62] Waizenegger J, Braeuning A, Templin M, Lampen A, Hessel-Pras S (2018) Structure-dependent induction of apoptosis by hepatotoxic pyrrolizidine alkaloids in the human hepatoma cell line HepaRG: single versus repeated exposure. Food Chem Toxicol 114:215–226. 10.1016/j.fct.2018.02.036 29458164 10.1016/j.fct.2018.02.036

[CR63] WHO (1988) WHO IPCS International Programme on Chemical Safety: Pyrrolizidine alkaloids Environmental Health Criteria, vol 80. World Health Organization, Geneva

[CR64] Wiedenfeld H, Röder E, Bourauel T, Edgar J (2008) Pyrrolizidine Alkaloids. V&R Unipress, Bonn, Germany

[CR65] Willmot FC, Robertson GW (1920) Senecio disease, or cirrhosis of the liver due to senecio poisoning. Lancet 196(5069):848–849. 10.1016/S0140-6736(01)00020-4

[CR66] Wilson DW, Lamé MW, Dunston SK, Segall HJ (2000) DNA damage cell checkpoint activities are altered in monocrotaline pyrrole-induced cell cycle arrest in human pulmonary artery endothelial cells. Toxicol Appl Pharmacol 166(2):69–80. 10.1006/taap.2000.8966 10896848 10.1006/taap.2000.8966

[CR67] Wu J, Lu LY, Yu X (2010) The role of BRCA1 in DNA damage response. Protein Cell 1(2):117–123. 10.1007/s13238-010-0010-5 21203981 10.1007/s13238-010-0010-5 PMC3078634

[CR68] Xiao Z, Xue J, Sowin TJ, Rosenberg SH, Zhang H (2005) A novel mechanism of checkpoint abrogation conferred by Chk1 downregulation. Oncogene 24(8):1403–1411. 10.1038/sj.onc.1208309 15608676 10.1038/sj.onc.1208309

[CR69] Yang X, Wang H, Ni HM et al (2017) Inhibition of Drp1 protects against senecionine-induced mitochondria-mediated apoptosis in primary hepatocytes and in mice. Redox Biol 12:264–273. 10.1016/j.redox.2017.02.020 28282614 10.1016/j.redox.2017.02.020 PMC5344326

