# Supplementary material for: The pyrrolizidine alkaloid lasiocarpine impairs cell cycle progression in vitro
Source: Arch Toxicol. 2025 Sep 16;99(12):4955–71. doi: 10.1007/s00204-025-04185-y (PMC12534242; doi:10.1007/s00204-025-04185-y)
Supplement: Supplementary file 2 — Supplementary material 2 [file 204_2025_4185_MOESM2_ESM.docx]

**- Supplemental Information -**

**The pyrrolizidine alkaloid lasiocarpine impairs cell cycle progression *in vitro***

Stefanie Hessel-Pras^a^, Marlena Beckschulte^b/^*, Antonia Peters^b/^*, Anja Koellner^b^, Beatrice Rosskopp^a^, Aaron Stahl^c^, Markus Templin^c^, Heike Sprenger^a^, Linda Boehmert^b^, Jan-Heiner Kuepper^d^, Benjamin Sachse^b^, Bernd Schaefer^b^

* equally contributed to this work

^a^ German Federal Institute for Risk Assessment, Department of Chemical and Product Safety, Max-Dohrn-Str. 8-10, 10589 Berlin, Germany

^b^ German Federal Institute for Risk Assessment, Department of Food and Feed Safety in the Food Chain, Max-Dohrn-Str. 8-10, 10589 Berlin, Germany

^c^ NMI Natural and Medical Sciences Institute, Department of Assay Development, Markwiesenstraße 55, 72770 Reutlingen, Germany

^d^ Brandenburg University of Technology (BTU) Cottbus-Senftenberg, Department of Molecular Cell Biology, Postfach 101344, 03013 Cottbus, Germany

Corresponding author:

Dr. Stefanie Hessel-Pras (ORCID: 0000-0002-6153-0035),

German Federal Institute for Risk Assessment

Max-Dohrn-Straße 8-10

10589 Berlin, Germany

e-mail: stefanie.hessel-pras@bfr.bund.de

Tel: +49 30 18412 25203


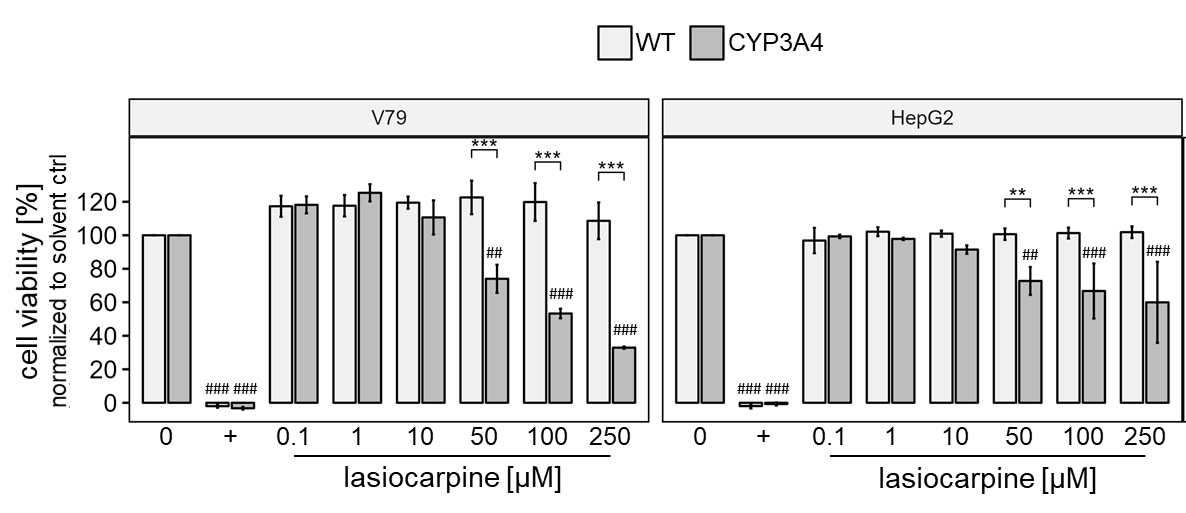


**Fig. S1** Cell viability in cultures of V79_WT_ and HepG2_WT_ cell lines and their respective derivatives overexpressing human CYP3A4 incubated with lasiocarpine for 24 h. Cells were treated with solvent (0.1% ACN, indicated by “0” in the figure), 0.01% Triton X-100 (+), and 0.1, 1, 10, 50, 100, or 250 μM lasiocarpine. Cell viability was determined by the neutral red uptake (NRU) assay. Means ± SD are shown from three single experiments with statistical significance levels compared to solvent control indicated by ^#^p < 0.05, ^##^p < 0.01, ^###^p < 0.001 and comparison between both cell lines is indicated by *p < 0.05, **p < 0.01, ***p < 0.001. Significance was tested by three-way ANOVA analysis followed by the estimated marginal means test as a post-hoc test


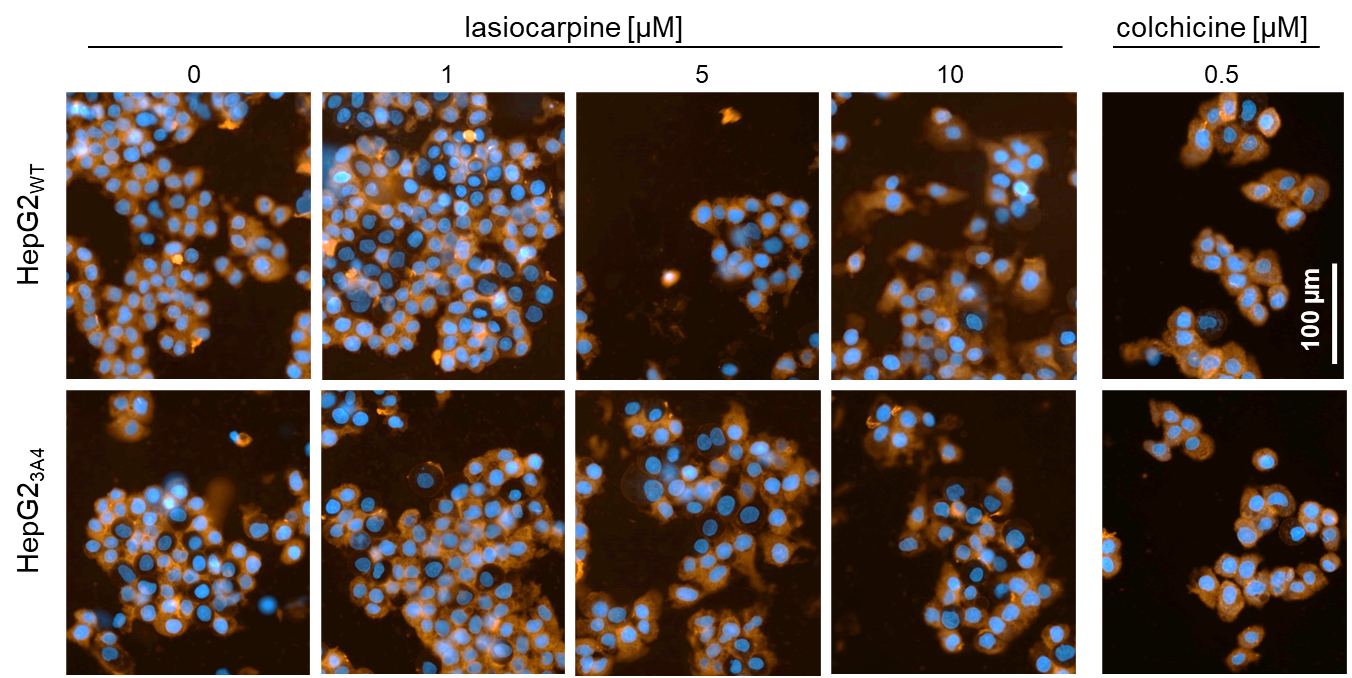


**Fig. S2** Examples of images representing lasiocarpine-induced effects on cell size of HepG2 cells and the respective CYP3A4-overexpressing cell line by Celldiscoverer 7 high content screening microscope. Cells were seeded as described in Material and Methods section. After treatment with lasiocarpine (1 to 10 µM), solvent (0 µM), or 0.5 µM colchicine serving as positive control for 24 h, nuclei and cell membrane were stained using Hoechst 33342 and CellMask™ Orange, respectively


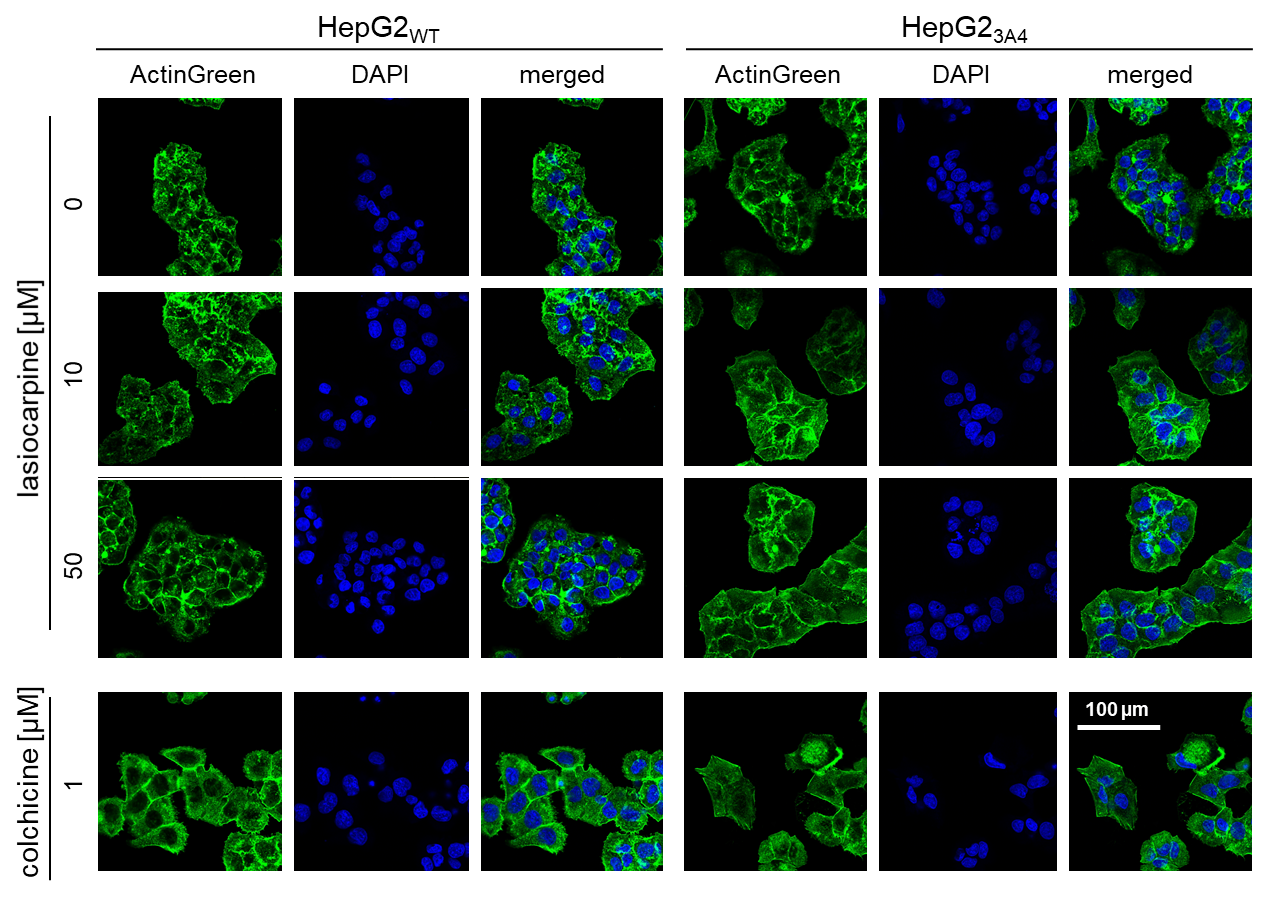


**Fig. S3** Representative confocal laser scanning microscopy images of synchronous HepG2_WT_ and HepG2_3A4_ cells. After synchronization with thymidine, cells were incubated with 1, 5 and 10 µM lasiocarpine. As controls, cells were treated with solvent (0.1% ACN/ 0 µM), or 0.5 μM colchicine as positive control for cell cycle disruption. Cells were fixed on glass coverslips, permeabilized and stained with ActinGreen™ 488 and DAPI as described in detail in Material and Methods section. Images were taken with Zeiss LSM 700 in a magnification of 40x

**
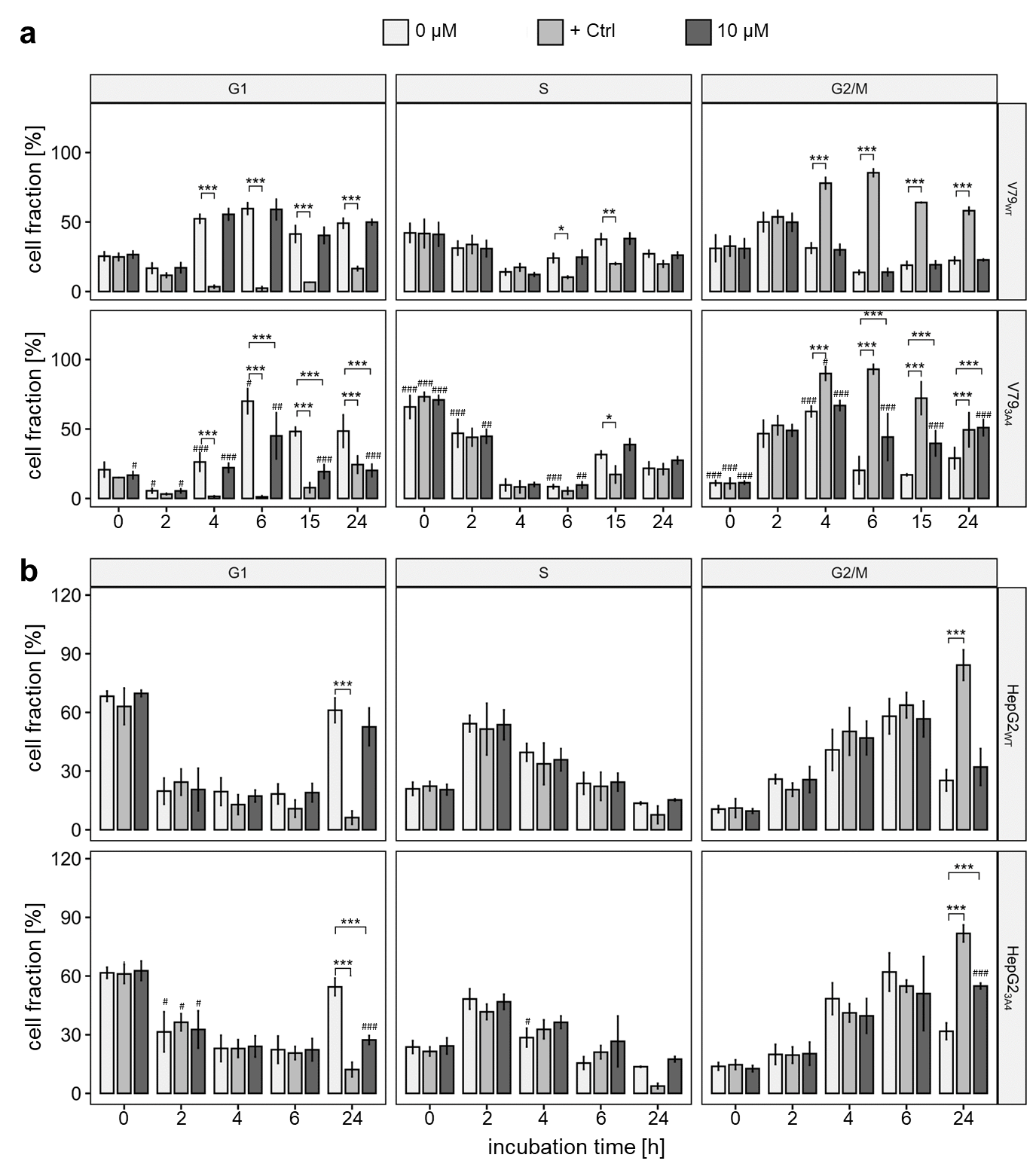
**

**Fig. S4** Cell cycle analysis of V79_WT_ and HepG2_WT_ and their respective derivatives overexpressing human CYP3A4 after treatment of cultures with thymidine before exposure to 10 μM lasiocarpine (Lc) for 0, (15), or 24 h. As controls, cells were treated with solvent (0 µM, medium + 0,1% ACN), or 1 μM colchicine (+ Ctrl) as a positive control for cell cycle disruption. Cell cycle analysis was performed by staining of DNA with propidium iodide (PI) and detecting the cell cycle phase by flow cytometry. Data are shown as means ± SD from three single experiments with statistical significance levels compared to solvent control indicated by *p < 0.05, **p < 0.01, ***p < 0.001 and comparison between both cell lines is indicated by ^#^p < 0.05, ^##^p < 0.01, ^###^p < 0.001. Significance was evaluated by three-way ANOVA analysis followed emmeans (estimated marginal means) test as post-hoc test


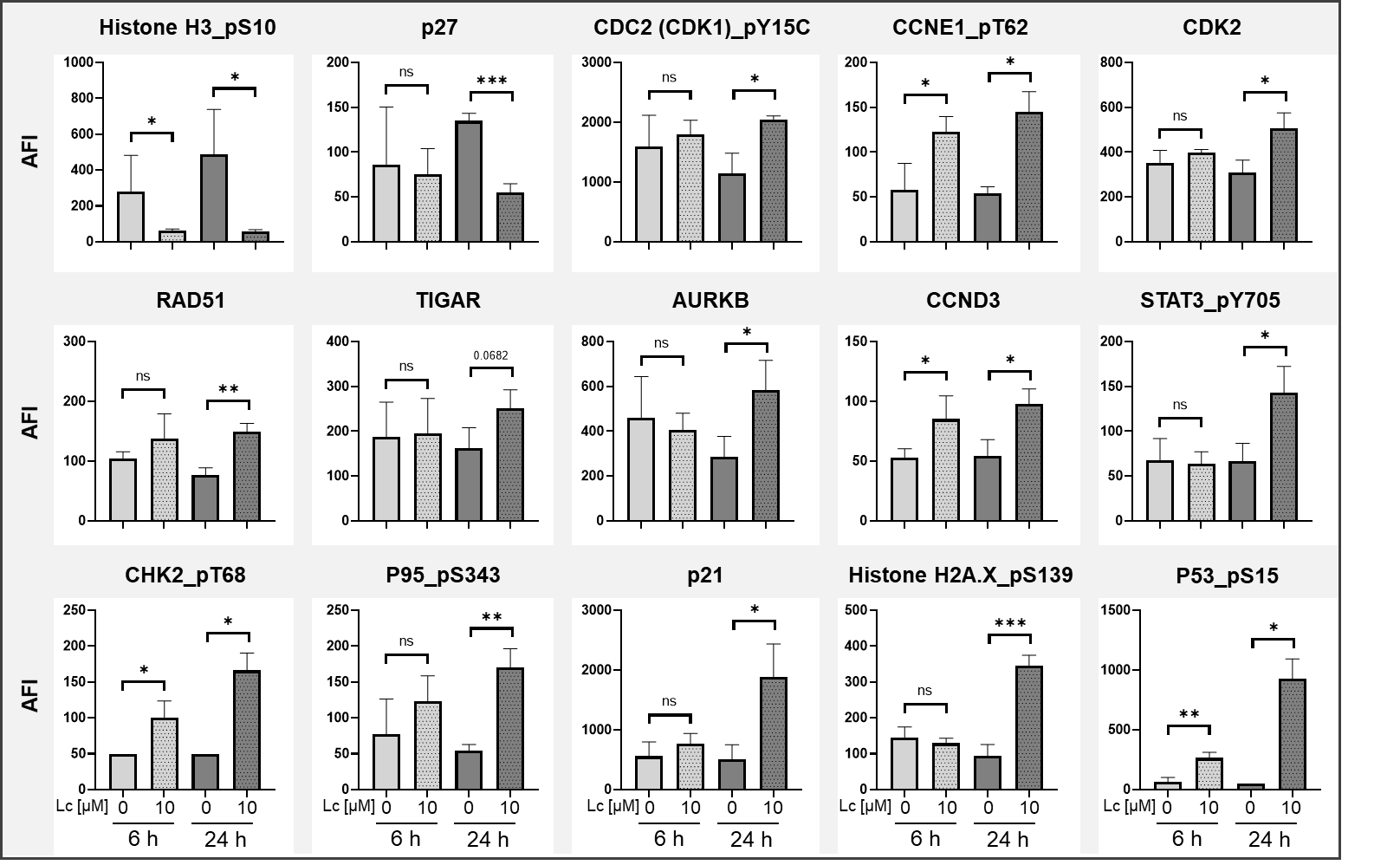


**Fig. S5** Representative bar graphs of significantly phosphorylated cell cycle and DNA damage response-associated proteins affected by lasiocarpine as analyzed by the DigiWest profiling assay. Synchronized HepG2_3A4_ cells were treated with 10 µM lasiocarpine or solvent (0.1% ACN/0 µM) for 6 or 24 h. For protein phosphorylation analysis, protein samples of three independent experiments were prepared and analyzed as described in the Material and Methods section. Mann-Whitney U-Test was used for comparison between groups using the accumulated fluorescence intensity (AFI) values. Statistical significance is indicated with * < 0.05, **p < 0.01, ***p < 0.001. ns, not significant
